# Naturally-Occurring Canine Mammary Tumors as a Translational Model for Human Breast Cancer

**DOI:** 10.3389/fonc.2020.00617

**Published:** 2020-04-28

**Authors:** Mark Gray, James Meehan, Carlos Martínez-Pérez, Charlene Kay, Arran K. Turnbull, Linda R. Morrison, Lisa Y. Pang, David Argyle

**Affiliations:** ^1^The Royal (Dick) School of Veterinary Studies and Roslin Institute, University of Edinburgh, Edinburgh, United Kingdom; ^2^Translational Oncology Research Group, Cancer Research UK Edinburgh Center, Institute of Genetics and Molecular Medicine, University of Edinburgh, Edinburgh, United Kingdom

**Keywords:** canine mammary cancer, comparative oncology, human breast cancer, *in vivo* models, translational models

## Abstract

Despite extensive research over many decades, human breast cancer remains a major worldwide health concern. Advances in pre-clinical and clinical research has led to significant improvements in recent years in how we manage breast cancer patients. Although survival rates of patients suffering from localized disease has improved significantly, the prognosis for patients diagnosed with metastatic disease remains poor with 5-year survival rates at only 25%. *In vitro* studies using immortalized cell lines and *in vivo* mouse models, typically using xenografted cell lines or patient derived material, are commonly used to study breast cancer. Although these techniques have undoubtedly increased our molecular understanding of breast cancer, these research models have significant limitations and have contributed to the high attrition rates seen in cancer drug discovery. It is estimated that only 3–6% of drugs that show promise in these pre-clinical models will reach clinical use. Models that can reproduce human breast cancer more accurately are needed if significant advances are to be achieved in improving cancer drug research, treatment outcomes, and prognosis. Canine mammary tumors are a naturally-occurring heterogenous group of cancers that have several features in common with human breast cancer. These similarities include etiology, signaling pathway activation and histological classification. In this review article we discuss the use of naturally-occurring canine mammary tumors as a translational animal model for human breast cancer research.

## Introduction

Cancer is a disease that occurs throughout the world, creating widespread social and economic burdens. Although advances in pre-clinical and clinical research are improving cancer diagnosis and treatment, the disease remains a significant cause of death throughout the world. In 2018 it was estimated that ~18 million new cancer cases were diagnosed and nearly 10 million cancer-related deaths occurred (1). Diagnostic and treatment approaches for cancer patients are constantly evolving, particularly in recent years due to increased interest in precision medicine (2). This concept uses disease biomarkers, phenotypes, molecular signatures, lifestyle and the environment to classify individual patients according to their differences in disease susceptibility, treatment responses and prognosis, ultimately allowing us to identify cohorts of patients that are more likely to respond to specific treatments and improve clinical outcomes (3). In order to achieve the goals of precision medicine and enable a move away from the traditional one-size-fits-all approach to cancer management we need to continually improve our understanding of cancer biology during its development and progression. This review will discuss the advantages of employing naturally-occurring canine mammary tumors as a translational animal model for human breast cancer and how their use can improve breast cancer research.

### Human Breast Cancer Subtypes

Human breast cancer (HBC) is a genetically and clinically heterogeneous disease (4). Breast cancer is the most commonly diagnosed cancer in women, with ~2 million new cases and 600,000 deaths occurring worldwide in 2018 (1). Survival rates continue to improve due to early diagnosis, advancements in surgical techniques and through the use of targeted therapies. Estimates of 5-year survival rates are ~97% for stage I, 88% for stage II and 70% for stage III (disease confined to local breast tissue or regional lymph nodes) (5). Despite advances in HBC management, the survival for metastatic stage IV disease (disease identified within distant organs or lymph nodes) remains poor at ~25% (5). Following a diagnosis of breast cancer, classification systems based on histological grading or molecular subtyping are commonly used to account for tumor heterogeneity, providing predictive and prognostic information which can influence a patient's treatment plan (6).

Histological classification systems categorize HBC into invasive and *in situ* carcinomas. Carcinomas *in situ* (CIS) are breast cancers in which malignant cells proliferate but remain confined within the basal membrane of the breast's terminal duct lobular units. These cancers can be sub-classified as either lobular or ductal (LCIS or DCIS). DCIS is the most common presentation and is typically characterized by the expression of E-cadherin. DCIS can be classified into 5 specific architectural subtypes (comedo, cribiform, micropapillary, papillary and solid) (7). Histological classification of DCIS and LCIS can be further evaluated by grading the expression levels of progesterone receptor (PR), estrogen receptor (ER), epidermal growth factor receptor 2 (ErbB2/HER2) (8), epidermal growth factor receptor (EGFR) and p53 (9–11). Invasive carcinomas can be histologically classified as invasive lobular, infiltrating ductal, ductal/lobular, tubular, mucinous, papillary and medullary (6). Infiltrating ductal carcinoma is the most common presentation of these subtypes, accounting for 70–80% of all invasive lesions (12). Assessment of ER, PR and HER2 expression, often referred to as receptor status, in invasive carcinomas provides an insight into the likely drivers of the disease and can determine the use of targeted therapies for specific cohorts of patients (11, 13). Examples of drug selection based on receptor status would include the use trastuzumab for HER2^+^ patients and aromatase inhibitors or tamoxifen for ER^+^/PR^+^ patients (6, 14–16).

Molecular classification systems aim to predict a tumors response to specific therapies. Largely based on microarray-based gene expression analysis, several intrinsic HBC molecular subtypes have been identified which have been shown to differ in treatment responses and predict overall survival (OS) and disease-free survival (DFS) (17–20). These subtypes include normal breast-like, HER2^+^, luminal A, luminal B, “claudin-low” and basal (17–19, 21). The ER^+^ subtypes (luminal A and luminal B) differ in clinical outcomes and patient survival. This molecular stratification is an important consideration, as clinical assessment of infiltrating ductal carcinomas using ER, PR, and HER2 receptor status cannot separate the two ER^+^ subtypes (6). Despite the predictive abilities of this molecular classification, its reliance on genome sequencing or microarray analysis means it remains cost-prohibitive for use in clinical practice. To overcome this obstacle, several studies have identified smaller gene sets which can be used to classify HBC into subtypes and predict prognosis or response to treatment. These clinically-available tests include the OncotypeDX 21-gene recurrence score (22), the BreastOncPx 14-gene distant metastasis signature (23) and the MammaPrint 70-gene prognosis signature (24). A 50-gene signature called PAM50 which has been shown to classify HBC into subtypes has improved the ability to predict recurrence of ER^+^/lymph node^−^ patients compared to models using only clinical variables (25, 26).

### Human Breast Cancer Treatment

Treatment of HBC can be administered either locally or systemically, depending largely on factors such as subtype and stage of disease. Typically, early and locally-advanced HBC is treated with surgery followed by adjuvant radiotherapy (RT), chemotherapy and/or endocrine therapy. RT with curative intent or as palliative treatment is estimated to benefit up to 83% of patients (27). Studies have also shown that whole-breast RT following breast-conserving surgery (BCS) provides local control and survival rates equivalent to mastectomy (28–30), with added advantages of improved cosmetic outcomes and reduced side effects. RT treatment plans typically involve the delivery of radiation to the tumor site in multiple fractions over a period of several weeks; the standard adjuvant RT fractionation regimen following BCS is 25 fractions of 2 Gy over 5 weeks, or hypofractionated regimens consisting of a total of 40 Gy delivered in 15 fractions over 3 weeks (31). Although overall 5-year survival rates after RT are ~80%, unfortunately around 30% of these patients will develop metastatic disease or local recurrence. It is these patients that have extremely poor 5 year survival rates (32).

Chemotherapy involves the administration of cytotoxic drugs that damage cellular targets such as DNA and tubulin in replicating cells. Many different chemotherapy drugs are available for use in HBC which can be given alone or in combination (cyclophosphamide, epirubicin, 5-flurouracil, methotrexate, doxorubicin, docetaxel, gemcitabine). Unfortunately, these drugs can cause significant side effects such as vomiting, alopecia, weight loss, fatigue, and immunosuppression (33). As a result of these wide-ranging side effects, treatments are usually given in cycles over a period of between 1–5 days, followed by a break of up to 4 weeks. Up to 8 treatment cycles may be given to breast cancer patients (34). Chemotherapy can be given prior to surgery (neoadjuvantly) to shrink the tumor before surgery (35) or after surgery (adjuvantly) in an attempt to prevent metastasis or recurrence. Adjuvant chemotherapy has been shown to improve overall patient survival by ~10% (36).

Various types of hormone treatments are available for ER^+^ breast cancers depending on whether the patient has gone through menopause or not. These therapies aim to block either the synthesis of the hormone estrogen or prevent its binding to ER. Prior to the menopause estrogen is produced primarily in the ovaries; this synthesis can be prevented either surgically by removing the ovaries (oophorectomy) or with endocrine therapies such as the gonadotropin-releasing hormone agonist goserelin (Zoladex™). However, the most common therapeutic tools used in the pre-menopausal setting are anti-oestrogens targeting the ER. Selective estrogen receptor modulators (SERMs), such as tamoxifen, are commonly used (37); they work by blocking the ER, thereby removing the growth-promoting effects of estrogen on the tumor. Fulvestrant (Faslodex™) is an example of a selective estrogen receptor down regulator (SERD) that both inhibits ER and accelerates its degradation (38). In post-menopausal women estrogen is synthesized from androgens in fat cells, through a reaction catalyzed by the enzyme aromatase (39). Letrozole (Femara™), anastrozole (Arimidex™) and exemestane (Aromasin™) are examples of aromatase inhibitors that work either by competing with substrates binding to aromatase, or by mimicking the substrates of aromatase and inactivating the enzyme. The overall result is to reduce the amount of estrogen produced and thus inhibit tumor growth (39). Post-menopausal women can also be treated with SERMs or SERDs to block the effect of the ER in cancer cells.

Targeted therapies have been developed to disrupt signaling pathways that are over-expressed or dysregulated in certain breast cancers (40). HER2 protein overexpression or gene amplification occurs in up to 34% of invasive breast cancers (41–43), leading to activation of numerous signaling pathways that are associated with cell growth and proliferation (44). Prognosis for patients presenting with HER2-overexpressing breast tumors has improved significantly since the advent of treatments targeting this receptor. Trastuzumab (Herceptin™) and Pertuzumab (Perjeta™) are monoclonal antibodies that bind to HER2 (45, 46) and block the cellular signaling pathways initiated by the receptor, thereby inhibiting the growth and survival of HER2-dependant tumors (47). Trastuzumab can be given in neoadjuvant, adjuvant and metastatic settings, and can improve OS of HER2-overexpressing breast cancer patients (48). Lapatinib (Tykerb™), a dual tyrosine kinase inhibitor of HER2 and EGFR, is another example of targeted therapy in clinical use (49). Both trastuzumab and lapatinib have been combined either together (50, 51) or with chemotherapy (52, 53) or hormone therapy (52, 54), leading to improved patient outcomes compared to single agent treatment. Other forms of targeted therapy involving trastuzumab include trastuzumab emtansine (Kadcyla™), which consists of a monoclonal antibody combined to a chemotherapeutic drug. With this treatment, trastuzumab directs the conjugate to the HER2-overexpressing breast cancer cells, inhibiting HER2 signaling, while the emtansine causes cancer cell death through impeding microtubule assembly (55). Another instance of a targeted therapy in clinical use is everolimus (Afinitor™); this drug inhibits the mammalian target of rapamycin (mTOR) pathway, which is involved in the regulation of the cell cycle, thereby inhibiting cancer cell growth (56). Palbocicib (Ibrance™), Ribociclib (Kisqali™), and Abemaciclib (Verzenios™) are selective inhibitors of the cyclin-dependent kinases CDK4 and CDK6 and are one of the newer classes of drugs to gain clinical approval for use in HBC patients. In post-menopausal women with ER^+^/HER2^−^ advanced breast cancer palbocicib can increase progression-free survival times when given in combination with letrozole compared with using letrozole alone (57).

### Limitations in Current Pre-clinical Cancer Research

**Figure 1:**
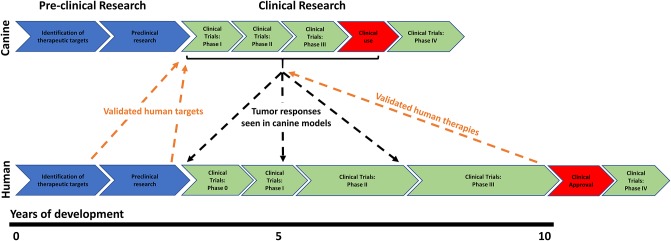
Schematic diagram outlining the integration of human and canine research programmes to improve current drug development strategies. The typical course of human drug research and development is very linear, progressing through *in vitro* target identification and validation right through to phase 0, I, II, III, and IV clinical trials. Unfortunately, most drugs which show promise in pre-clinical studies subsequently fail to show efficacy in clinical trials. At this point a substantial amount of time and money has been spent, which can ultimately deter drug companies from developing new therapeutics. The integration of translational canine studies into human drug development programmes could help identify drug failures sooner or allow for drug refinement prior to human clinical studies. Shorter disease-free progression times seen in dogs also allows for rapid conclusion of the clinical trials that can incorporate assessment of drug activity, toxicity, pharmacokinetics, pharmacodynamics, dose regimens, combination therapies and histology.

In the United States of America it is currently estimated that drug development from inception to regulatory approval requires ~13 years and between $1.8–2.6 billion (58); despite this enormous investment, between 86–95% of drugs fail to show efficacy or gain approval for use. The majority of agents fail during clinical trials, after a considerable amount of time and money has already been invested in drug development (58, 59). The situation is even worse for the development of new cancer drugs, where only 3–6% of drugs reach clinical use (59). There are numerous limitations associated with traditional pre-clinical studies which tend to focus on cancer cells grown in 2D or 3D cultures (60) or murine xenograft models (61, 62) to assess the efficacy of cancer agents; these limitations have contributed to these high drug attrition rates. One method by which traditional drug development strategies could be improved is to integrate translational pre-clinical animal models into the drug development process at an early stage. These models provide an opportunity to evaluate all aspects of drug development, ranging from efficacy, pharmacokinetics/dynamics and toxicity assessment, through to formulating dosing schedules. These studies could be completed before drugs are taken into more expensive and time-consuming human clinical studies. The early discovery of drug failures would allow drug refinement prior to human clinical studies and ultimately reduce the failure rates observed in these trials (Figure 1).

### Canine Comparative Oncology

Comparative oncology enables the investigation of human cancers by using similar cancers that occur naturally in animals. The dog is an excellent example of comparative oncology, as the species develops numerous tumors that have similar clinicopathological features or incidence rates to specific human cancers (63–65). Risk factors involved in carcinogenesis are also similar between the two species, including environmental toxins (dogs and humans will be exposed to similar carcinogens as they share a common living environment), obesity and advancing age (66). The advantages of studying cancers in this species over current pre-clinical models are substantial and are largely due to the tumors developing naturally in the presence of a functioning immune system (34).

The popularity of keeping dogs as pets has meant that there are ~12 million pet dogs in the UK (67); this large population therefore provides a valuable resource opportunity. The shorter lifespan of dogs compared to humans enables researchers to study cancers that develop after a few years instead of decades, a time course that is sufficient to allow comparison of treatment responses while still being short enough to ensure rapid conclusion of the clinical trials. Dogs are also more comparable with humans than rodents in terms of size, anatomy, physiology, metabolism, immunology and genetics. Sequencing of the canine genome (99% complete, ~2.5 billion base pairs) (68) has shown that there are greater similarities between dog and human gene sequences than compared with mice (69). Procedures that are commonly used in human medicine such as sample collection, surgery and imaging can be easily used in dogs but are harder to apply to rodent models. For these reasons dogs are increasingly being regarded as an excellent model for use in translational cancer research and have been shown to be a highly-predictive model for drug development in humans (63, 65, 70).

### Canine Mammary Cancer

Canine mammary tumors (CMT) are the most commonly diagnosed cancer in female dogs; accounting for almost 50% of all canine neoplasms (71). The incidence rate per year is ~198 cases in every 100,000 dogs (66); this rate is higher than that seen in humans, where the annual incidence of HBC is 85 cases per 100,000 women (1). Taking into account the different lifespans of the two species, the highest incidence of mammary tumor development is similar between dogs (8–11 years) and humans (50–58 years); this rate has been shown to increase with age, with the disease rarely diagnosed before 5 and 25 years of age in dogs and humans, respectively (72–76).

The hormonal etiology of CMT has been well-established and has largely been based on an initial study looking at the incidence of CMT in intact and spayed (ovariohysterectomised) female dogs. This study found that CMT developed in ~0.5% of female dogs that were spayed before their first season, with levels rising to 8 and 26% when dogs were spayed either after their first or second season. Furthermore, spaying dogs following their second season had no protective effect on the risk of developing malignant mammary tumors (77). Diet and obesity have also been shown to affect CMT incidence rates. One study showed that if dogs were classified as thin at 9–12 months of age, they had a significantly reduced risk for mammary tumor development compared with control dogs (the risk was reduced by 99 and 40% in spayed and intact dogs, respectively) (78). A further study found that dogs given a diet high in red meat and dogs that were obese at 1 year old were at an increased risk of developing mammary dysplasia and tumors (79). In post-menopausal women, obesity is suggested to be a risk factor for breast cancer development through raising circulating estrogen levels and increasing local estrogen production by aromatases (80, 81). It is possible that obesity escalates the risk for mammary tumors in dogs through similar mechanisms, especially as both canine studies indicated that obesity has the greatest effect on mammary tumor development if present early in a dog's life. This may be due to hormones having the most damaging effects on developing mammary tissue at this stage in a dog's life.

### Histology of Canine Mammary Cancer

Histological diagnosis remains the most common method for classifying CMT. Periodic reviews have led to modifications of the original histological classification system first published in 1974 (82). One such modification was the World Health Organization international classification system, published in 1999, which combined histology, morphology and prognostic information (Table 1) (83); further refinement was brought about through advances in understanding CMT biology. These results subsequently led to the recognition and incorporation of new histologic subtypes into the classification system (84).

**Table 1:** The different types of malignant and benign canine mammary tumors based on the WHO classification system [adapted from Misdorp et al. (83)].

**Histological classification**	**Description**
**MALIGNANT MAMMARY TUMORS**	
**Carcinomas**	
*In situ* carcinoma	No invasion of the basement membrane
Complex carcinoma	Presence of luminal epithelial and myoepithelial components
Simple carcinoma:	Composed of one cell type, resembling either luminal epithelial, or myoepithelial cells. Often invasive with lymphatic and/or haematogenous spread. Increasing malignancy from tubulopapillary to solid to anaplastic tumors
*Tubulopapillary carcinoma*	Characterized by tubules and/or papillary projections
*Solid carcinoma*	Characterized by the arrangement of tumor cells in solid sheets, cords or nests
*Anaplastic carcinoma*	Characterized by highly infiltrative pleomorphic epithelial cells
Spindle cell carcinoma	Spindle cells arranged in epithelial patterns
Squamous cell carcinoma	Characterized by solid sheets and cords of cells with squamous differentiation
Mucinous carcinoma	Characterized by mucin production
Lipid-rich carcinoma	Characterized by cells with vacuolated cytoplasm containing large amounts of lipids
**Sarcomas**	
Fibrosarcoma	Fibroblasts with collagen production
Osteosarcoma	Characterized by neoplastic cellular osteoid and/or bone formation
Chondrosarcoma	Very rare
Liposarcoma	Very rare
**Mixed**	
Carcinosarcoma	Carcinomatous and sarcomatous components
Carcinoma/sarcoma in benign tumors	Foci of malignant cells within a complex adenoma or benign mixed tumor
**BENIGN MAMMARY TUMORS**	
**Adenomas**	
Simple	Well differentiated luminal epithelial or myoepithelial cellular tumor
Complex	Characterized by luminal epithelial and myoepithelial cells
Basaloid	Uniform cords and clusters of basaloid epithelial cells
**Fibroadenomas**	
Low/high cellularity	Mixed luminal epithelial cells, stromal cells and myoepithelial cells
**Mixed**	
Benign	Benign cells resembling epithelial components (luminal and/or myoepithelial) and mesenchymal cells that have produced cartilage, bone or fat in combination with fibrous tissue

Grading systems, such as the Elston and Ellis method are also commonly used to provide prognostic information by providing a malignancy score (85). CMT can also occur in multiple glands at the same time with 60% of tumors located with the caudal glands. CMT can also have substantial histological variation both within a single specific tumor and between different tumors in the same dog (86, 87). CMT can be purely of epithelial (simple adenoma or simple carcinoma) or mesenchymal (fibroadenoma, fibrosarcoma, osteosarcoma, or other sarcomas) origin; however, some consist of a combination of epithelial and myoepithelial tissues (complex adenoma or complex carcinoma) or epithelial and mesenchymal tissues (benign mixed tumors or carcinosarcoma) (84). Approximately 50% of all CMT are malignant (71, 88), the majority of which are carcinomas.

Inflammatory mammary carcinoma is a rare and specific form of CMT (89, 90). The disease has a similar incidence rate to the equivalent disease in HBC, accounting for ~7% and 5% of all canine and human mammary tumors, respectively (89, 91). Inflammatory mammary carcinoma is locally invasive with genetic, biological and clinical characteristics that differ from the other forms of mammary cancers (89, 92) and is histologically diagnosed by invasion of dermal lymphatic vessels by neoplastic emboli (93).

### Canine Mammary Tumors and Metastatic Disease

Depending on the subtype diagnosis, CMT can be fatal due to the development of metastatic disease. Studies have shown that 50% of canine mammary carcinomas metastasise to local lymph nodes. Lymph node involvement invariably leads to distant metastases, which is seen most commonly in the lung, although metastatic bone lesions can also occur (94, 95). This clinical course is similar to HBC, where ~7% of women will present with metastatic disease and 20% of those with local disease will eventually develop metastatic lesions (96). Although research has identified several prognostic markers such as age, tumor size, local/distant metastases, clinical stage and histological subtype that can separate human and canine patients into cohorts that have greater recurrence or mortality risk (Table 2) (98, 102, 105–107, 112–114), a detailed understanding of the molecular mechanisms which influence how these cancers metastasise remains unclear.

**Table 2:** Canine mammary tumor prognostic factors (DFS, disease-free survival; OS, overall survival) [adapted from Sleeckx et al. (97)].

**Factor**	**Effect on prognosis**
Age	Increased age at diagnosis can reduce DFS and OS (98–101)
Tumor size	Increased tumor size can reduce DFS and OS (99–103). Dogs with a tumor size of 3–5 cm can have a median OS of 22 months; whereas dogs with tumors >5 cm can have a median OS of 14 months
Skin ulceration	Presence of skin ulceration can reduce DFS and OS (98, 99, 101)
Histological subtype	Subtype classification can correlate with prognosis (83, 99, 101, 103, 104): Anaplastic carcinomas can have a median OS of 2.5 months; solid carcinomas can have a median OS of 16 months, whereas tubulopapillary carcinomas can have a median OS of 21 months
Tumor stage	Tumor stage at diagnosis correlates with OS (103, 104). Stage I: median OS of 24 months; Stage II: median OS of 12 months; Stage III: median OS of 15 months; Stage IV and V: median OS of 6 months
Grade	Tumor grade at diagnosis correlates with 2-year survival (99, 101, 105). Grade I: 100%; grade II: 53.3%; grade III: 13.5%
Lymph node metastasis	The presence of lymph node metastasis at diagnosis correlates with 2-year survival (98, 99, 101, 103–107). 85% of dogs without lymph node metastasis will be alive 2 years post-surgery, compared with only 21% that have lymph node metastasis
Distant metastasis	The presence of distant metastasis at diagnosis correlates with OS (103, 106). Dogs without metastases at time of surgery can have median OS of 28 months compared with only 5 months for dogs with metastasis
Expression of ER and PR	Low expression correlates with reduced DFS and OS (102, 106, 107)
Expression of Cox-2	High expression correlates with reduced DFS and OS (108–111)
Expression of proliferation markers	High expression correlates with increased risk of metastasis and reduced DFS and OS (98, 101, 106, 107)

To investigate the value of CMT as a metastatic model, studies have investigated both gene (115) and protein (116) signatures identified in metastatic CMT and compared them with equivalent human samples. Metastatic CMT were shown to have ~1,000 genes that were significantly differentially expressed compared to non-metastatic carcinomas. Up-regulated genes were associated with cell cycle regulation, DNA damage repair, matrix modulation, protein folding and proteasomal degradation. These results are similar to a meta-analysis study using metastatic HBC expression profiles which found significant up-regulation of cell cycle and DNA replication pathways. Down-regulated genes were associated with cell differentiation, growth factor pathways, focal adhesion pathways and regulators of actin organization. Of the differentially expressed canine genes, 25% were found to be associated with HBC; expression profiles of metastatic CMT also contained parts of a human prognostic gene signature (115).

Similar results to those observed at gene level have been identified at the protein level (116). One study identified 21 proteins with significant changes in expression between metastasising and non-metastasising CMT; these proteins were predominantly associated with cellular functions related to metastasis including extracellular matrix remodeling, cell adhesion and resistance to hypoxia. Higher expression levels of proteins involved with proliferation (such as proliferating cell nuclear antigen) and cell motility (tropomyosin 3, Coronin 1A, adenosine deaminase) were also identified in metastasising compared with non-metastasising carcinomas. The same study also showed that metastatic cells had increased protein expression of free radical scavengers, which may protect the cells from hypoxic and oxidative stress caused by rapid tumor growth and poor vascularisation. While the expression of thioredoxins had not been previously investigated in CMT, increased expression levels have been found in poorly differentiated human colorectal and hepatocellular carcinomas. Literature searches using the 21 differentially expressed proteins in the metastatic CMT showed that 19 of them had previously been associated with either malignancy or metastasis in a variety of human cancers. Furthermore, 9 of the proteins had a similar malignancy-associated protein expression pattern as seen in HBC.

The partially overlapping transcriptome and proteome of metastatic CMT and HBC suggests that there are underlying comparable mechanisms involved in mammary carcinogenesis and pathogenesis between the 2 species and provides evidence that metastatic canine carcinomas are a suitable translational model for human breast tumors which could be used to determine prognostic and predictive molecular signatures and identify therapeutic targets. Furthermore, the differential gene and protein expression profiles in metastatic and non-metastatic carcinomas could predict prognosis and identify molecular pathways and networks involved in CMT metastasis.

### Molecular Markers

The use of molecular markers, such as hormone or signaling receptors in HBC diagnosis has had a major influence on patient treatment regimens. Markers have the potential to predict which patients will most likely respond to certain treatments and provide prognostic information. Although these markers are not routinely used in veterinary medicine, increasing evidence suggests that these human-derived molecular markers might be similarly useful for CMT evaluation.

#### Hormones

Steroid hormones (estrogen and progesterone) and their receptors play significant roles in HBC development. As in women, CMT are predominantly hormone-dependent; this is evidenced by the fact that, as with early pregnancy/oophorectomy in women, early spaying in dogs is linked with lower disease incidence. ER and PR have also been identified in ~70% of benign and 60% of malignant CMT (117–119); this is similar to that seen in HBC, where ~60–70% of all tumors contain these receptors (120–122). In humans, ER and PR are useful prognostic indicators; ER expression for example is known to be associated with increased DFS and OS of breast cancer patients (119). ER expression is also a predictor of tumor hormone dependency and thus response to endocrine therapy (123–125). Several studies have now shown that, like in humans, ER and PR expression can also be prognostic and predictive in CMT, with a decrease in hormonal dependency commonly seen in malignant tumors (122, 126). Normal mammary gland tissue, mammary dysplasia, and benign tumors have been shown to have higher ER expression levels than malignant tumors (118, 127), with a recent study demonstrating that dogs with ER^−^/PR^+^ tumors had significantly worse survival rates in comparison to dogs with ER^+^/PR^+^ tumors. Dogs with ER^−^/PR^−^ tumors had the most guarded prognosis of all (122). Higher ER expression levels have also been shown to occur in the tumors of young dogs, those that remain genitally intact and in dogs that have regular oestrous cycles (107). Metastatic lesions are also frequently ER^−^/PR^−^ and malignant PR^−^ tumors have been shown to proliferate at higher rates than PR^+^ tumors. A further study has also shown that ER expression is lower in malignant compared with benign tumors, with lower expression also identified in larger tumors and in those where skin ulceration was present (107). Lymph node involvement and the development of distant metastasis was also associated with low ER expression levels. Proliferation, assessed by proliferating cell nuclear antigen, was found to be negatively correlated with ER expression levels, supporting results seen in HBC that well-differentiated tumors can maintain a hormonal regulation of cell division leading to lower proliferation rates. This study also showed that ER expression, Ki-67 index, and age were predictors of DFS, and that age, lymph node status and ER expression were all prognostic for OS. These results have been supported by a large study of 350 dogs diagnosed with malignant carcinomas in which tumor size larger than 20 mm, positive lymph node, a histological grade III, ER^−^ status and high Ki-67 proliferation index were all prognostic factors for poor OS. Overall, these studies indicate that molecular markers, especially ER, in CMT can be used as prognostic indicators and predictors of DFS and OS and could be useful in selecting appropriate hormonal therapy as is the case in HBC (106).

#### Signaling Receptors and Associated Pathways

As described earlier, HER2 is an intensively studied proto-oncogene in HBC which functions to promote tumor growth, cellular differentiation and survival. Approximately 30% of all HBC cases express HER2, this subtype has high metastatic potential and carries a poor prognosis (41–43). HER2 protein overexpression is both prognostic for disease outcome and predictive for the response to targeted therapies (128) and in the vast majority of cases results from HER2 gene amplification (129). Fluorescence *in situ* hybridization (FISH) is a sensitive technique for evaluating HER2 gene status in terms of gene amplification and copy number which may be superior to IHC HER2 assessment. One study showed that patients classified as HER2 positive by FISH but negative by IHC had poorer survival rates than those who had HER2 overexpression in the absence of gene amplification (130). Chromogenic *in situ* hybridization (CISH) is an alternative technique to FISH which has been shown to be an accurate, practical, and economical approach to determining HER2 status in HBC (129). In veterinary medicine, investigations into CMT HER2 expression have not been as abundant as compared with that in human medicine. Several studies have identified either HER2 protein or gene expression in ~35% of malignant CMT (131–134); however these studies did not investigate the simultaneous analysis of HER2 protein and gene status. One study has investigated the relationship between HER2 gene and protein expression in CMT through the use of CISH and IHC. HER2 protein overexpression was found in 17.6% of the carcinomas tested; however, these cases were not associated with gene amplification. The authors suggested that CMT could be translational models of HBC where HER2 overexpression occurs in the absence of gene amplification (135). HER2 expression has been correlated with mitotic index, histological grade and tumor size (131, 133, 136). Although HER2 expression is thought to be associated with poor prognosis (137) some studies have shown no difference in HER2 expression between benign and malignant tumors (132, 138, 139). It has therefore been suggested that HER2 expression may be involved in carcinogenesis but may not play a significant role in malignant transformation; as yet, its role as a marker of malignancy in CMT undetermined. Through genetic sequencing it has been shown that there is a high degree of homology between the human and canine HER2 antigens; this has led to the suggestion that human antibody-based immunotherapies such as trastuzumab or pertuzumab could be utilized in HER2 expressing CMT (140).

EGFR has been highlighted as a potential prognostic molecular marker and possible therapeutic target in triple negative HBC (ER^−^/PR^−^/HER2^−^) (141). In CMT high EGFR expression has been associated with increased angiogenesis, large tumor size, tumor necrosis, higher mitotic rates, advanced clinical stage and malignancy (142–144). However, its role in mammary carcinogenesis or prognosis is unclear. Although studies have shown a tendency toward shorter DFS and OS for dogs with CMT expressing EGFR, these associations have failed to reach statistically significant levels (143). Downstream pathways of EGFR have also been implicated in HBC pathogenesis; results from the first genome-wide comparative analysis of canine and human mammary tumors showed up-regulation of numerous pathways related to increased proliferation, whereas pathways involved with cell development, communication and matrix adhesion were down-regulated. This study also demonstrated significant homology between canine and human cancers in terms of changes to known cancer-related signaling pathways including KRAS, PI3K/AKT, MAPK, PTEN and Wnt-β-catenin (145). The PI3K/AKT pathway is a very well-studied pathway that is involved in regulating cellular transformation, proliferation and survival. In HBC, genetic aberrations can lead to PI3K/AKT pathway activation through the presence of activating point mutations at phosphoinositide-3-kinase, catalytic, alpha polypeptide (*PIK3CA*), mutation of v-akt-murine thymoma viral oncogene homolog 1 (*AKT1*) and loss of phosphatase and tensin homolog (*PTEN*) activity (146–151). Active PI3K/AKT signaling has been identified in CMT in association with a decrease in expression of the tumor suppressor gene *PTEN*. Loss of *PTEN* has been suggested as a prognostic marker in CMT (145, 152, 153).

E-cadherin is an important membrane bound cell adhesion molecule and tumor suppressor gene which is commonly expressed in epithelial tissues. Loss of E-cadherin can occur in HBC and CMT and is associated with tumor size, histological grade, stage of disease and prognosis (154–156). Loss of E-cadherin is also a key feature of epithelial-mesenchymal transition (EMT), which can promote metastasis through increasing a cell's ability to migrate and invade (157). Another member of the cadherin family that appears to be involved in HBC pathogenesis is P-cadherin. P-cadherin is expressed by mammary tissue myoepithelial cells and has been found to be overexpressed in high-grade invasive breast carcinomas. Expression is associated with poor prognosis as the molecule is known to enhance cell invasion and tumor aggressiveness (158). Similar results have been found in CMT, with higher P-cadherin expression levels being associated with malignant histologic subtypes and increased invasive properties (154).

The *p53* tumor suppressor gene plays a major role in the prevention of cellular malignant transformation, acting through its control of the cell cycle, cell growth, DNA repair mechanisms, apoptosis and autophagy (159). Through these mechanisms, p53 can influence a cells fate following DNA damage and can confer resistance to tumourigenesis in human mammary epithelial cells. Conversely, *p53* gene mutations can lead to deregulated cell proliferation and tumourigenesis. These aberrations are more commonly found in triple negative HBC and high expression in this subtype correlates with poor prognosis (160). The frequency of *p53* mutations in CMT is ~20%, which is similar to that seen in HBC (161–163). In HBC, accumulation of p53 nuclear protein, resulting from *p53* gene mutation, has been shown to be correlated with poor OS (162, 164). In CMT, *p53* gene mutations and protein overexpression are considered predictors of malignancy and poor prognosis (163, 165, 166).

Although only a limited number of molecular markers have been highlighted here, there are a significant number of others which have been investigated for their similarities between HBC and CMT. These include insulin-like growth factor, growth hormone, Wnt signaling, mucins, heat shock proteins, CEA, CA 15–3, VEGF and cyclooxygenases (137, 167). The similarities observed between mammary tumors occurring in dogs and humans in terms of target genes and molecular signaling pathways provides significant evidence that this canine cancer model can be regarded as a homolog for human cancer biology. Research into CMT could facilitate cross-species development of pathway-targeted therapeutic agents, identification of prognostic or predictive biomarkers and evaluation of drug responses.

### Canine Mammary Cancer Subtypes

Subtype classification of CMT has been investigated in a number of studies which typically use immunohistochemical expression of various cellular markers. One large study used PR, ERα, HER2, EGFR, Ki-67, and CK5/6 and as the molecular markers. Evaluation of their expression levels identified several distinct subtypes including luminal A (14.3%), luminal B (9.4%), triple-negative basal-like (58.6%), and triple-negative non-basal-like (17.7%), although no HER2-overexpressing CMT were observed (168). A further study based CMT classification on HER2 and ER expression which identified luminal A (ER^+^/HER2^−^, 44.8%), luminal B (ER^+^/HER2^+^, 13.5%), basal-like (ER^−^/HER2^−^, 29.2%) and HER2-overexpressing (ER^−^/HER2^+^, 8.3%) subtypes (169). The PAM50 human classification system has also been used to subtype CMT. This analysis was repeated 100 times, ensuring that each TCGA tumor was sampled at least once. Notably, in 82 of 100 times, all canine simple carcinomas and ER complex carcinoma (ID 518) groups clustered with the human basal-like tumors. The remaining canine complex carcinomas (all ERβ), however, failed to cluster with any specific human subtypes. This study explained these results were due to the canine tumors not possessing HER2 overexpression or amplification. Furthermore, consistent with the basal-like subtype a portion of these canine mammary carcinomas also harbored copy number abnormalities and were ER^−^, with DNA repair and cell cycle related genes significantly up-regulated. Basal-like HBC are inherently aggressive which carry a poor prognosis. Currently there are no effective therapies which can be used against this subtype. However, studies such as the ones described here have provided evidence for the use of canine mammary carcinomas as a model to study basal-like HBC. This model therefore could provide translational information toward understanding and treating this breast cancer subtype (170). Although there appears to be some variation in the specific subtypes and their incidence levels identified in each study, overall the results suggest that subtype classification is associated with prognosis; luminal A subtypes exhibit significantly longer DFS and OS compared to triple-negative carcinomas. The results from these CMT studies demonstrate the molecular heterogeneity of the disease and highlight the models use for studying breast cancer carcinogenesis and pathogenesis.

### BRCA1, BRCA2 Mutations

Several genes, including *CHEK2, HER2, RCAS1, TP53, FGFR2, LSP1, MAP3K1*, and *TOX3* are known to increase the risk of HBC (171–174). The development of breast cancer is thought to be polygenic, where tumourigenesis is influenced by numerous loci, each contributing a small risk to disease development (175, 176). However, the most commonly studied genes are *BRCA1* and *BRCA2* (*BRCA1/2*). The *BRCA1/2* genes are members of the granin family. They function as tumor suppressors playing critical roles in maintaining genome stability largely through regulating transcription and controlling the DNA damage response, DNA repair and cell cycle (177). Mammary tumor development in dogs and women has been associated with deregulation of *BRCA1/2* gene function (145, 178, 179). *BRCA1/2* germline mutations account for ~5–10% of all HBC cases (180, 181), with inherited *BRCA1/2* mutations increasing the risk of breast cancer up to 84% (182–184). Most *BRCA1/2* gene mutations occur through non-sense mutations, indels, rearrangements, or splice variants which cause the proteins to become truncated (185, 186). *BRCA2* is commonly overexpressed and associated with poor prognosis in HBC, whereas decreased *BRCA1* expression is frequently observed during disease progression (187–189). The histology of breast cancers in women predisposed by *BRCA1* and *BRCA2* mutations differs in several ways. *BRCA2* and *BRCA1* mutations have also been associated with male breast cancer and ovarian cancer, respectively. When *BRCA1* breast tumors are compared with that of sporadic cases they are more likely to show higher grades of malignancy, be classified as ER^−^ and PR^−^ and carry a worse prognosis (190).

As dogs have a long history of inbreeding with low levels of genetic variation, it has been suggested that CMT development in a single breed should have a more defined homogenous origin compared with that of HBC, which occurs within a diverse and much larger population. In theory this concept should allow for the identification of breed specific CMT risk factors. One study investigated the development of CMT in English springer spaniels and identified that the *BRCA1/2* genes were associated with a 4-fold increase in the risk of developing CMT (191). However, this study did not look at gene mutations and further investigations are required to understand them and identify functional mechanisms, i.e., how these genes influence tumourigenesis. A more recent CMT study has shown that *BRCA2* messenger RNA expression decreases in adenomas but increases in mammary carcinoma lymph node metastases when compared with non-neoplastic mammary epithelium (192). A reduction in the nuclear expression of *BRCA1* has been shown to occur in CMT which was associated with high proliferation index and loss of ER. These findings were more significantly associated with malignant phenotypes (193). These studies provide further evidence that CMT is an excellent model for HBC with a specific focus on a genetic hereditary component of the disease.

### Mammography and Ultrasound Imaging

Screening procedures and diagnosis of HBC commonly involves the use of ultrasonography and x-ray mammography (194). X-ray mammograms can be useful to diagnose DCIS through the identification of microcalcifications which can be present in up to 72% of cases (194, 195). Ultrasound and X-ray mammography have been investigated in canine pre-invasive and invasive mammary tumors to assess whether the disease has similarities to HBC in terms of imaging appearance (196). Canine mammary mammographic and ultrasonographic abnormalities in pre-invasive lesions, benign and malignant tumors were found to be similar to those commonly seen in HBC, including the presence and distribution pattern of microcalcifications and macrocalcifications. The identification of calcification was also associated with malignancy. This study indicated that CMT form similar microcalcifications and macrocalcifications to HBC and suggested that a comparable carcinogenesis process may be occurring between the two species. Furthermore, the authors concluded that sonographic and mammographic characteristics of benign and malignant CMT could be associated with histopathological diagnosis and that mammographic categorization was therefore a precise method for the detection of malignant CMT.

### Treatment and Prognosis

As in human cancer patients, disease staging for CMT is mandatory before beginning definitive treatment. Staging would typically include blood work and thoracic and/or abdominal radiographs and cytologic evaluation (87, 97). Surgery with either lumpectomy or radical mastectomy remains the most widely accepted treatment option for CMT (197). One large prospective study found no significant difference in local recurrence rates or OS between dogs receiving either a simple lumpectomy or radical mastectomy (86); however, a further smaller study identified that 58% of dogs which received local removal of individual mammary tumors developed new tumors in the remaining ipsilateral mammary glands, suggesting more radical surgery may be required (198). Concurrent ovariohysterectomy may also improve survival rates for ER^+^ tumors and those dogs with raised serum estrogen levels (199). In one recent study involving 350 dogs diagnosed with invasive mammary carcinomas, OS after mastectomy was only 11 months (106). Chemotherapy is commonly used to treat triple negative or metastatic HBC and, although only limited studies have been conducted for its use in the treatment of CMT, various chemotherapeutic agents are commonly used in dogs to treat a variety of other cancers (97). A small case study has shown that post-operative 5-fuorouracil and cyclophosphamide did improve DFS and OS in dogs with malignant mammary tumors compared with dogs that received only surgery (200). Inflammatory mammary carcinomas have an extremely poor survival rate, with no effective treatment. Multimodal therapy, including neoadjuvant chemotherapy and/or RT before mastectomy has improved DFS and OS times in women with the condition. One study has investigated the use of chemotherapy (mitoxantrone or mitoxantrone combined with cyclophosphamide and vincristine) in addition to palliative treatment of dogs with inflammatory carcinomas. Although the study did show that chemotherapy improved survival times from 35 to 57 days, the prognosis remained extremely poor (93). Hormonal therapy is a commonly used treatment option in humans with ER^+^ breast cancer. In dogs however the use of anti-oestrogens, such as tamoxifen, for the treatment of CMT has only been reported in a limited number of clinical studies. The results of these canine studies have also provided conflicting results (201, 202). Anti-estrogen therapy can also cause significant side effects in dogs which includes vulvar swelling, vaginal discharge, pyometra (intact females) and stump pyometra (spayed females) (202, 203). RT is seldom used in the treatment of CMT but could be considered to aid local disease control for incompletely resected tumors or as a palliative treatment for non-resectable or inflammatory mammary carcinomas. Further investigations to determine the role of RT in the treatment of CMT are required because of the significant role it plays in the treatment of HBC (197). These studies provide evidence that the potentially aggressive nature of CMT, especially in the case of invasive and inflammatory carcinomas, can allow clinical trials to be completed in shorter time frames than human trials would allow. Rapid generation of data could then be used to aid the design of human trials, potentially improving the selection of drugs taken forward (106).

### Limitations and Achievements Associated With the use of Naturally-Occurring Canine Cancer Models for Translational Research

Although comparative oncology is not a new concept, the idea of integrating canine studies of naturally-occurring cancers into the drug development process is still not commonly practiced. Lack of model awareness and poorly coordinated infrastructures between veterinary hospitals, oncologists, researchers and pharmaceutical companies are important factors which have held back the potential of using such a model (63). Understanding the risks and challenges that are preventing the use of these naturally-occurring canine cancer models is essential if we are to overcome some of the limitations associated with traditional drug development strategies.

Researchers might consider that the increased time and monetary investments required to complete canine studies, compared with resources typically needed in murine models, are major limiting factors for their use. Study costs and detailed budgets are mandatory requirements for canine studies and will need to include factors such as drug production (the larger size of dogs compared to mice will mean larger drug quantities are required), recruitment of the required number of dogs, obtaining clinical resources and data analysis. Ultimately, the cost of a canine study will depend on the specific scientific question that needs to be answered and its defined clinical endpoint; these important factors must be established first as they will dictate the design of the trial. The addition of diagnostic procedures, tumor biopsies, imaging, follow-up assessments, sample collection and processing all incrementally add to study costs (65). However, it must be stressed that these types of procedures add significant value to the drug development process and can produce data which is difficult or impossible to obtain using murine models. If an integrated canine model approach is successful at prioritizing drugs for development and use in humans, then these study costs will be minor and offset by the substantial cost reductions seen in subsequent human clinical trials.

Other important factors that researchers need to consider if dogs are to be used pre-clinically is that the studies must be conducted using standard guidelines with ethical approval and informed owner consent. The lack of readily available protocols might deter researchers from using the model. Results from such studies should be reported in a timely manner. In the USA, an electronic reporting system, developed by the Comparative Oncology Trials Consortium (COTC), allows data to be acquired in real-time and provides a means by which study results can be monitored (65); unfortunately, systems such as this are not yet widely available.

A significant advantage of using CMT for HBC research is that the disease is the most commonly diagnosed cancer in female dogs, with an incidence rate greater than that seen in the human population (1, 66, 71). Although there is potentially a large cohort of dogs that could be recruited into studies, a significant limitation of using this canine population for HBC research is that the current standard of care treatment options between the 2 species differ significantly. As described earlier in the review, regardless of tumor subtype (excluding inflammatory mammary carcinomas) surgical resection alone is the most commonly used treatment option for CMT. This contrasts with HBC, where subtype can influence treatment and typically consists of patients receiving multimodal therapy. These differences in treatment regimes can make the generation of a translational clinical trial difficult. Recruitment of CMT patients into a clinical trial may also have challenges. Although there may be a large population of dogs suitable for recruitment, informed owner consent is required before enrolment into the trial, which can be difficult to obtain. Currently, there is a lack of patient stratification for those dogs receiving surgery alone; therefore, justification for inclusion into trails investigating adjuvant therapies may be difficult. Ultimately these CMT studies may therefore be restricted to dogs receiving palliative treatment, those that failed standard treatments or those which are unfit for surgery. However, recruiting these dogs may be difficult as owners will typically opt for euthanasia due to poor prognosis. Increasing owner awareness by providing evidence of why canine studies can help both veterinary and medical communities would help overcome these issues and improve patient recruitment.

One of the accepted advantages of using murine cancer models, including breast, is that the tumors they produce are genetically homogenous and as such, tumors in different mice will respond in similar ways to a specific drug (204). Subtle effects can be more easily identified, and animal sample sizes can therefore be reduced. Although in this review we have shown that CMT are similar to HBC, the tumors that occur in both species, even those classified as the same subtype, can have considerable heterogeneity. Even though clinical trials can be specifically tailored to a specific tumor subtype, this inherent mammary tumor heterogeneity will ultimately result in a variation of tumor responses to drugs. This may require increased numbers of dogs to be recruited and larger sample sizes to be obtained. However, it is these types of trials, that incorporate tumor heterogeneity, that will ultimately provide a more translational and clinically-relevant model that will improve drug development strategies.

An excellent example of the use of naturally-occurring canine cancers in a phase I clinical trial involved the investigation of the safety and efficacy of a novel receptor tyrosine kinase (RTK) inhibitor, SU11654 (205). This synthetic compound was developed to inhibit multiple members of the split-kinase domain family of RTKs, including platelet derived growth factor receptor (PDGFR), vascular endothelial growth factor receptor (VEGFR), FLT3, and KIT. Phase I trials were undertaken as SU11654 had shown anti-proliferative and anti-angiogenic activity in *in vitro* studies and in *in vivo* murine xenograft experiments (206). Fifty-seven dogs over a 1-year period that were diagnosed with a variety of naturally-occurring tumors, including mammary carcinomas, which had failed conventional therapy or for which no therapeutic alternative was available were recruited into the study. As these dogs had a very poor prognosis other selection criteria such as estimated life expectancy of >6 weeks and adequate organ function were included into the recruitment process to allow the study to run over a long enough time period to allow toxicity and tumor response data to be obtained. Results showed that the greatest tumor response rates were seen in mast cell tumors (MCT) and that dogs with MCT possessing KIT mutations were much more likely to respond to therapy than those tumors with wild-type KIT. Aberrant KIT signaling in MCT can be due to activating mutations, similar to those found in gastro-intestinal stromal tumors in humans, consisting of internal tandem duplications in the juxtamembrane domain of KIT which results in constitutive receptor phosphorylation. In terms of the 5 mammary carcinoma cases included in the study, serial thoracic CT scans identified regression of pulmonary metastases in 2 cases (partial responders: 21 weeks and >60 weeks) and no change in size of pulmonary metastases in a further 2 cases (stable disease: 27 and 38 weeks). Although this study did not investigate the mode of action of SU11654 in these CMT cases, aberrant VEGFR, PDGFR, and KIT expression has been identified in HBC (207–209) with high KIT expression levels having also been identified in CMT (210). The authors suggested that given the similarities between canine and human cancers in terms of tumor biology and the presence of analogous RTK dysregulation, SU11654 or similar compounds could elicit comparable biological responses in both species. Further studies confirmed the benefits of using SU11654 in canine MCT (211) and toceranib (Palladia™) became the first dog-specific anti-cancer drug to gain clinical approval.

Following the success of these trials a sister compound to toceranib called sunitinib (Sutent™), was later clinically approved for the treatment of renal cell carcinoma and gastrointestinal stromal tumors in human patients; however, its role in the treatment of HBC patients remains open to debate. One recent systematic review evaluated the efficacy of using sunitinib alone or in combination with chemotherapy for the treatment of advanced HBC and suggested that sunitinib produced no clinical benefit. However, the authors suggested that further studies that stratify patients based on perhaps molecular markers are warranted to fully ascertain sunitinib's use in HBC (212).

Although this canine phase I clinical trial is just one example, it provides evidence of how the early integration of naturally-occurring canine cancers into the drug development process can be implemented successfully and demonstrates how the previously described limitations can be overcome.

## Conclusions

Naturally-occurring CMT have significant potential for use as a model to study various aspects of HBC biology. The disease in both species shares similar etiology, histopathological classification and pathogenesis, with known oncogenic drivers such as HER2 and estrogen signaling. The shorter lifespan of dogs and aggressive nature of the disease means that clinical trials involving new therapeutic agents can be completed far quicker and cheaper than human phase O-IV studies. The results from the integration of CMT patients into clinical trials as pre-clinical models could feed back on the design of subsequent human trails. The aim of using translational models in this way would be to reduce the high rates of failure currently seen in human proof-of-concept studies. This concept would ultimately save time and money. The successful adoption of the translational aspects of the CMT model into cancer research would not only improve our molecular understanding of breast cancer, but also improve pre-clinical research and ultimately the treatment of canine and human breast cancer patients.

## Author Contributions

MG, JM, LP, and DA conceptualized the article. MG wrote the majority of the manuscript. Figure and tables were composed by MG, JM, LM, CM-P, CK, and AT. Critical revisions were made by MG, JM, CM-P, CK, AT, LM, LP, and DA. All authors read and approved the final manuscript.

## Conflict of Interest

The authors declare that the research was conducted in the absence of any commercial or financial relationships that could be construed as a potential conflict of interest.

## References

[1] Bray F Ferlay J Soerjomataram I Siegel RL Torre LA Jemal A. Global cancer statistics 2018: GLOBOCAN estimates of incidence and mortality worldwide for 36 cancers in 185 countries. CA Cancer J Clin. (2018) 68:394–424. 10.3322/caac.21492 30207593

[2] Ghasemi M Nabipour I Omrani A Alipour Z Assadi M. Precision medicine and molecular imaging: new targeted approaches toward cancer therapeutic and diagnosis. Am J Nuclear Med Mol Imaging. (2016) 6:310–27. 28078184 PMC5218860

[3] Penet MF Krishnamachary B Chen Z Jin J Bhujwalla ZM. Molecular imaging of the tumor microenvironment for precision medicine and theranostics. Adv Cancer Res. (2014) 124:235–56. 10.1016/B978-0-12-411638-2.00007-0 25287691 PMC4332825

[4] Stingl J Caldas C. Molecular heterogeneity of breast carcinomas and the cancer stem cell hypothesis. Nat Rev Cancer. (2007) 7:791–9. 10.1038/nrc2212 17851544

[5] Macia F Porta M Murta-Nascimento C Servitja S Guxens M Buron A. Factors affecting 5- and 10-year survival of women with breast cancer: an analysis based on a public general hospital in Barcelona. Cancer Epidemiol. (2012) 36:554–9. 10.1016/j.canep.2012.07.003 22854422

[6] Malhotra GK Zhao X Band H Band V. Histological, molecular and functional subtypes of breast cancers. Cancer Biol Ther. (2010) 10:955–60. 10.4161/cbt.10.10.13879 21057215 PMC3047091

[7] Connolly J Recommendations for the reporting of breast carcinoma. Pathol Case Rev. (1998) 3:241–7. 10.1097/00132583-199809000-00006

[8] Poller DN Silverstein MJ Galea M Locker AP Elston CW Blamey RW. Ideas in pathology. Ductal carcinoma *in situ* of the breast: a proposal for a new simplified histological classification association between cellular proliferation and c-erbB-2 protein expression. Mod Pathol. (1994) 7:257–62. 7911998

[9] Holland R Peterse JL Millis RR Eusebi V Faverly D van de Vijver MJ. Ductal carcinoma *in situ*: a proposal for a new classification. Semin Diagn Pathol. (1994) 11:167–80. 7831528

[10] Silverstein MJ Poller DN Waisman JR Colburn WJ Barth A Gierson ED. Prognostic classification of breast ductal carcinoma-in-situ. Lancet. (1995) 345:1154–7. 10.1016/S0140-6736(95)90982-6 7723550

[11] Gradishar WJ Anderson BO Balassanian R Blair SL Burstein HJ Cyr A. Invasive breast cancer version 1.2016, NCCN clinical practice guidelines in oncology. J Natl Compr Cancer Netw. (2016) 14:324–54. 10.6004/jnccn.2016.0181 26957618

[12] Li CI Uribe DJ Daling JR. Clinical characteristics of different histologic types of breast cancer. Br J Cancer. (2005) 93:1046–52. 10.1038/sj.bjc.6602787 16175185 PMC2361680

[13] Harris L Fritsche H Mennel R Norton L Ravdin P Taube S. American Society of Clinical Oncology 2007 update of recommendations for the use of tumor markers in breast cancer. J Clin Oncol. (2007) 25:5287–312. 10.1200/JCO.2007.14.2364 17954709

[14] Maughan KL Lutterbie MA Ham PS. Treatment of breast cancer. Am Fam Phys. (2010) 81:1339–46. 20521754

[15] Rakha EA Reis-Filho JS Ellis IO. Combinatorial biomarker expression in breast cancer. Breast Cancer Res Treat. (2010) 120:293–308. 10.1007/s10549-010-0746-x 20107892

[16] Payne S Bowen R Jones J Wells C. Predictive markers in breast cancer–the present. Histopathology. (2008) 52:82–90. 10.1111/j.1365-2559.2007.02897.x 18171419

[17] Perou CM Sorlie T Eisen MB van de Rijn M Jeffrey SS Rees CA. Molecular portraits of human breast tumours. Nature. (2000) 406:747–52. 10.1038/35021093 10963602

[18] Sorlie T Perou CM Tibshirani R Aas T Geisler S Johnsen H. Gene expression patterns of breast carcinomas distinguish tumor subclasses with clinical implications. Proc Natl Acad Sci USA. (2001) 98:10869–74. 10.1073/pnas.191367098 11553815 PMC58566

[19] Sorlie T Tibshirani R Parker J Hastie T Marron JS Nobel A. Repeated observation of breast tumor subtypes in independent gene expression data sets. Proc Natl Acad Sci USA. (2003) 100:8418–23. 10.1073/pnas.0932692100 12829800 PMC166244

[20] Sotiriou C Neo SY McShane LM Korn EL Long PM Jazaeri A. Breast cancer classification and prognosis based on gene expression profiles from a population-based study. Proc Natl Acad Sci USA. (2003) 100:10393–8. 10.1073/pnas.1732912100 12917485 PMC193572

[21] Prat A Parker JS Karginova O Fan C Livasy C Herschkowitz JI. Phenotypic and molecular characterization of the claudin-low intrinsic subtype of breast cancer. Breast Cancer Res. (2010) 12:68–75. 10.1186/bcr2635 20813035 PMC3096954

[22] Paik S Shak S Tang G Kim C Baker J Cronin M. A multigene assay to predict recurrence of tamoxifen-treated, node-negative breast cancer. N Engl J Med. (2004) 351:2817–26. 10.1056/NEJMoa041588 15591335

[23] Tutt A Wang A Rowland C Gillett C Lau K Chew K. Risk estimation of distant metastasis in node-negative, estrogen receptor-positive breast cancer patients using an RT-PCR based prognostic expression signature. BMC Cancer. (2008) 8:339. 10.1186/1471-2407-8-339 19025599 PMC2631011

[24] van de Vijver MJ He YD van't Veer LJ Dai H Hart AA Voskuil DW. A gene-expression signature as a predictor of survival in breast cancer. N Engl J Med. (2002) 347:1999–2009. 10.1056/NEJMoa021967 12490681

[25] Parker JS Mullins M Cheang MC Leung S Voduc D Vickery T. Supervised risk predictor of breast cancer based on intrinsic subtypes. J Clin Oncol. (2009) 27:1160. 10.1200/JCO.2008.18.1370 19204204 PMC2667820

[26] Ellis MJ Suman VJ Hoog J Lin L Snider J Prat A. Randomized phase II neoadjuvant comparison between letrozole, anastrozole, and exemestane for postmenopausal women with estrogen receptor–rich stage 2 to 3 breast cancer: clinical and biomarker outcomes and predictive value of the baseline PAM50-based intrinsic subtype—ACOSOG Z1031. J Clin Oncol. (2011) 29:2342. 10.1200/JCO.2010.31.6950 21555689 PMC3107749

[27] Delaney G Jacob S Featherstone C Barton M. The role of radiotherapy in cancer treatment: estimating optimal utilization from a review of evidence-based clinical guidelines. Cancer. (2005) 104:1129–37. 10.1002/cncr.21324 16080176

[28] Onitilo AA Engel JM Stankowski RV Doi SA. Survival comparisons for breast conserving surgery and mastectomy revisited: community experience and the role of radiation therapy. Clin Med Res. (2015) 13:65–73. 10.3121/cmr.2014.1245 25487237 PMC4504664

[29] Cao J Olson R Tyldesley S. Comparison of recurrence and survival rates after breast-conserving therapy and mastectomy in young women with breast cancer. Curr Oncol. (2013) 20:593–601. 10.3747/co.20.1543 24311961 PMC3851357

[30] Poortmans P. Evidence based radiation oncology: breast cancer. Radiother Oncol. (2007) 84:84–101. 10.1016/j.radonc.2007.06.002 17599597

[31] Haviland JS Owen JR Dewar JA Agrawal RK Barrett J Barrett-Lee PJ. The UK Standardisation of Breast Radiotherapy (START) trials of radiotherapy hypofractionation for treatment of early breast cancer: 10-year follow-up results of two randomised controlled trials. Lancet Oncol. (2013) 14:1086–94. 10.1016/S1470-2045(13)70386-3 24055415

[32] Allemani C Sant M Weir HK Richardson LC Baili P Storm H. Breast cancer survival in the US and Europe: a CONCORD high-resolution study. Int J Cancer. (2013) 132:1170–81. 10.1002/ijc.27725 22815141 PMC4706735

[33] Selwood K Side Effects of Chemotherapy. Cancer in Children and Young People. Hoboken, NY: John Wiley & Sons, Ltd (2009) p. 35–71. 10.1002/9780470988145.ch4

[34] Pang LY Argyle DJ. Using naturally occurring tumours in dogs and cats to study telomerase and cancer stem cell biology. Biochim Biophys Acta. (2009) 1792:380–91. 10.1016/j.bbadis.2009.02.010 19254761

[35] Untch M Konecny GE Paepke S von Minckwitz G. Current and future role of neoadjuvant therapy for breast cancer. Breast. (2014) 23:526–37. 10.1016/j.breast.2014.06.004 25034931

[36] Joerger M Thurlimann B. Chemotherapy regimens in early breast cancer: major controversies and future outlook. Expert Rev Anticancer Ther. (2013) 13:165–78. 10.1586/era.12.172 23406558

[37] Swaby RF Sharma CG Jordan VC. SERMs for the treatment and prevention of breast cancer. Rev Endocr Metab Disord. (2007) 8:229–39. 10.1007/s11154-007-9034-4 17440819

[38] Johnston SJ Cheung KL. Fulvestrant - a novel endocrine therapy for breast cancer. Curr Med Chem. (2010) 17:902–14. 10.2174/092986710790820633 20156170

[39] Brueggemeier RW. Update on the use of aromatase inhibitors in breast cancer. Expert Opin Pharmacother. (2006) 7:1919–30. 10.1517/14656566.7.14.1919 17020418

[40] Widakowich C de Castro G Jr de Azambuja E Dinh P Awada A. Review: side effects of approved molecular targeted therapies in solid cancers. Oncologist. (2007) 12:1443–55. 10.1634/theoncologist.12-12-1443 18165622

[41] Worzfeld T Swiercz JM Looso M Straub BK Sivaraj KK Offermanns S. ErbB-2 signals through Plexin-B1 to promote breast cancer metastasis. J Clin Invest. (2012) 122:1296–305. 10.1172/JCI60568 22378040 PMC3314465

[42] Gutierrez C Schiff R. HER2: biology, detection, and clinical implications. Arch Pathol Lab Med. (2011) 135:55–62. 10.1043/2010-0454-RAR.1 21204711 PMC3242418

[43] Ross JS Fletcher JA. HER-2/neu (c-erb-B2) gene and protein in breast cancer. Am J Clin Pathol. (1999) 112(1 Suppl. 1):53–67. 10396301

[44] Moasser MM. The oncogene HER2: its signaling and transforming functions and its role in human cancer pathogenesis. Oncogene. (2007) 26:6469–87. 10.1038/sj.onc.1210477 17471238 PMC3021475

[45] Gianni L Pienkowski T Im Y-H Roman L Tseng L-M Liu M-C Efficacy and safety of neoadjuvant pertuzumab and trastuzumab in women with locally advanced, inflammatory, or early HER2-positive breast cancer (NeoSphere): a randomised multicentre, open-label, phase 2 trial. Lancet Oncol. (2012) 13:25–32. 10.1016/S1470-2045(11)70336-9 22153890

[46] Schneeweiss A Chia S Hickish T Harvey V Eniu A Hegg R. Pertuzumab plus trastuzumab in combination with standard neoadjuvant anthracycline-containing and anthracycline-free chemotherapy regimens in patients with HER2-positive early breast cancer: a randomized phase II cardiac safety study (TRYPHAENA). Ann Oncol. (2013) 24:2278–84. 10.1093/annonc/mdt182 23704196

[47] Hudis CA. Trastuzumab–mechanism of action and use in clinical practice. N Engl J Med. (2007) 357:39–51. 10.1056/NEJMra043186 17611206

[48] Drakaki A Hurvitz SA HER2-positive breast cancer: update on new and emerging agents. Am J Hematol/Oncol. (2015) 11:17–23.

[49] Moreira C Kaklamani V. Lapatinib and breast cancer: current indications and outlook for the future. Expert Rev Anticancer Ther. (2010) 10:1171–82. 10.1586/era.10.113 20735304

[50] Blackwell KL Burstein HJ Storniolo AM Rugo H Sledge G Koehler M. Randomized study of Lapatinib alone or in combination with trastuzumab in women with ErbB2-positive, trastuzumab-refractory metastatic breast cancer. J Clin Oncol. (2010) 28:1124–30. 10.1200/JCO.2008.21.4437 20124187

[51] Blackwell KL Burstein HJ Storniolo AM Rugo HS Sledge G Aktan G. Overall survival benefit with lapatinib in combination with trastuzumab for patients with human epidermal growth factor receptor 2-positive metastatic breast cancer: final results from the EGF104900 Study. J Clin Oncol. (2012) 30:2585–92. 10.1200/JCO.2011.35.6725 22689807

[52] Hurvitz SA Kakkar R Role of lapatinib alone or in combination in the treatment of HER2-positive breast cancer. Breast Cancer. (2012) 4:35–51. 10.2147/BCTT.S29996 24367193 PMC3846547

[53] Valachis A Mauri D Polyzos NP Chlouverakis G Mavroudis D Georgoulias V. Trastuzumab combined to neoadjuvant chemotherapy in patients with HER2-positive breast cancer: a systematic review and meta-analysis. Breast. (2011) 20:485–90. 10.1016/j.breast.2011.06.009 21784637

[54] Kaufman B Mackey JR Clemens MR Bapsy PP Vaid A Wardley A. Trastuzumab plus anastrozole versus anastrozole alone for the treatment of postmenopausal women with human epidermal growth factor receptor 2-positive, hormone receptor-positive metastatic breast cancer: results from the randomized phase III TAnDEM study. J Clin Oncol. (2009) 27:5529–37. 10.1200/JCO.2008.20.6847 19786670

[55] Dieras V Bachelot T. The success story of trastuzumab emtansine, a targeted therapy in HER2-positive breast cancer. Target Oncol. (2014) 9:111–22. 10.1007/s11523-013-0287-4 23852665

[56] Barnett CM. Everolimus: targeted therapy on the horizon for the treatment of breast cancer. Pharmacotherapy. (2012) 32:383–96. 10.1002/j.1875-9114.2012.01084.x 22461124

[57] Finn RS Martin M Rugo HS Jones S Im S-A Gelmon K. Palbociclib and Letrozole in advanced breast cancer. N Engl J Med. (2016) 375:1925–36. 10.1056/NEJMoa1607303 27959613

[58] Schomberg DT Tellez A Meudt JJ Brady DA Dillon KN Arowolo FK Miniature swine for preclinical modeling of complexities of human disease for translational scientific discovery and accelerated development of therapies and medical devices. Toxicol Pathol. (2016) 44:299–314. 10.1177/0192623315618292 26839324

[59] Wong CH Siah KW Lo AW Estimation of clinical trial success rates and related parameters. Biostatistics. (2019) 20:273–86. 10.1093/biostatistics/kxx069 29394327 PMC6409418

[60] Kapałczynska M Kolenda T Przybyła W Zajaczkowska M Teresiak A Filas V. 2D and 3D cell cultures - a comparison of different types of cancer cell cultures. Arch Med Sci. (2018) 14:910–9. 10.5114/aoms.2016.63743 30002710 PMC6040128

[61] Shimosato Y Kameya T Hirohashi S. Growth, morphology, and function of xenotransplanted human tumors. Pathol Annu. (1979) 14:215–57. 94941

[62] Sharkey FE Fogh J. Considerations in the use of nude mice for cancer research. Cancer Metastasis Rev. (1984) 3:341–60. 10.1007/BF00051459 6394126

[63] Paoloni M Khanna C. Translation of new cancer treatments from pet dogs to humans. Nat Rev Cancer. (2008) 8:147–56. 10.1038/nrc2273 18202698

[64] Rowell JL McCarthy DO Alvarez CE. Dog models of naturally occurring cancer. Trends Mol Med. (2011) 17:380–8. 10.1016/j.molmed.2011.02.004 21439907 PMC3130881

[65] Gordon I Paoloni M Mazcko C Khanna C. The Comparative Oncology Trials Consortium: using spontaneously occurring cancers in dogs to inform the cancer drug development pathway. PLoS med. (2009) 6:e1000161. 10.1371/journal.pmed.1000161 19823573 PMC2753665

[66] Meuten DJ Tumors in Domestic Animals. Hoboken, NY: John Wiley & Sons (2016). 10.1002/9781119181200

[67] Murray J Gruffydd-Jones T Roberts M Browne W Assessing changes in the UK pet cat and dog populations: numbers and household ownership. Vet Record. (2015) 2015:103223 10.1136/vr.103223 26350589

[68] Lindblad-Toh K. Genome sequence, comparative analysis and haplotype structure of the domestic dog. Nature. (2005) 438:803–19. 10.1038/nature04338 16341006

[69] Hoffman MM Birney E. Estimating the Neutral Rate of Nucleotide Substitution Using Introns. Mol Biol Evol. (2006) 24:522–31. 10.1093/molbev/msl179 17122369

[70] Fenger JM Rowell JL Zapata I Kisseberth WC London CA Alvarez CE Dog models of naturally occurring cancer. Anim Model Hum Cancer. (2016) 153–221. 10.1002/9783527695881.ch9

[71] Moe L. Population-based incidence of mammary tumours in some dog breeds. J Reprod Fertil Suppl. (2001) 57:439–43. 11787188

[72] Pollán M Pastor-Barriuso R Ardanaz E Argüelles M Martos C Galcerán J. Recent changes in breast cancer incidence in Spain, 1980–2004. J Natl Cancer Inst. (2009) 101:1584–91. 10.1093/jnci/djp358 19861303 PMC2778670

[73] Dobson JM Samuel S Milstein H Rogers K Wood JL. Canine neoplasia in the UK: estimates of incidence rates from a population of insured dogs. J Small Anim Pract. (2002) 43:240–56. 10.1111/j.1748-5827.2002.tb00066.x 12074288

[74] Schneider R. Comparison of age, sex, and incidence rates in human and canine breast cancer. Cancer. (1970) 26:419–26. 5465470 10.1002/1097-0142(197008)26:2<419::aid-cncr2820260225>3.0.co;2-u

[75] Cohen D Reif JS Brodey RS Keiser H. Epidemiological analysis of the most prevalent sites and types of canine neoplasia observed in a veterinary hospital. Cancer Res. (1974) 34:2859–68. 4529096

[76] Salas Y Marquez A Diaz D Romero L. Epidemiological study of mammary tumors in female dogs diagnosed during the Period 2002–2012: a growing animal health problem. PLoS ONE. (2015) 10:e0127381. 10.1371/journal.pone.0127381 25992997 PMC4436381

[77] Schneider R Dorn CR Taylor D. Factors influencing canine mammary cancer development and postsurgical survival. J Natl Cancer Inst. (1969) 43:1249–61. 4319248

[78] Sonnenschein EG Glickman LT Goldschmidt MH McKee LJ. Body conformation, diet, and risk of breast cancer in pet dogs: a case-control study. Am J Epidemiol. (1991) 133:694–703. 10.1093/oxfordjournals.aje.a115944 2018024

[79] Alenza DP Rutteman GR Peña L Beynen AC Cuesta P Relation between habitual diet and canine mammary tumors in a case-control study. J Vet Int Med. (1998) 12:132–9. 10.1111/j.1939-1676.1998.tb02108.x 9595373

[80] Greenberg E Vessey M McPherson K Doll R Yeates D. Body size and survival in premenopausal breast cancer. Br J Cancer. (1985) 51:691–3. 10.1038/bjc.1985.104 3994912 PMC1977063

[81] Harvie M Howell A Vierkant RA Kumar N Cerhan JR Kelemen LE. Association of gain and loss of weight before and after menopause with risk of postmenopausal breast cancer in the Iowa women's health study. Cancer Epidemiol Biomarkers Prev. (2005) 14:656–61. 10.1158/1055-9965.EPI-04-0001 15767346

[82] Hampe J Misdorp W. Tumours and dysplasias of the mammary gland. Bull World Health Organ. (1974) 50:111–33. 4371737 PMC2481221

[83] Misdorp W Else R Hellmen E Lipscomb T Histological Classification of the Mammary Tumors of the Dog and the Cat. World Health Organization International Histological Classification of Tumors of Domestic Animals second series. Silver Spring, MD: American Registry of Pathology (1999) 7:1–59.

[84] Goldschmidt M Peña L Rasotto R Zappulli V. Classification and grading of canine mammary tumors. Vet Pathol. (2011) 48:117–31. 10.1177/0300985810393258 21266722

[85] Elston CW Ellis IO. Pathological prognostic factors in breast cancer. I. The value of histological grade in breast cancer: experience from a large study with long-term follow-up. Histopathology. (1991) 19:403–10. 10.1111/j.1365-2559.1991.tb00229.x 1757079

[86] dos Santos Horta R Lavalle GE de Castro Cunha RM de Moura LL de Araújo RB Cassali GD Influence of surgical technique on overall survival, disease free interval and new lesion development interval in dogs with mammary tumors. Adv Breast Cancer Res. (2014) 3:38–48. 10.7287/peerj.preprints.143

[87] Shafiee R Javanbakht J Atyabi N Kheradmand P Kheradmand D Bahrami A. Diagnosis, classification and grading of canine mammary tumours as a model to study human breast cancer: an Clinico-Cytohistopathological study with environmental factors influencing public health and medicine. Cancer Cell Int. (2013) 13:79. 10.1186/1475-2867-13-79 23937693 PMC3765114

[88] Sorenmo K. Canine mammary gland tumors. Vet Clin North Am Small Anim Pract. (2003) 33:573–96. 10.1016/S0195-5616(03)00020-2 12852237

[89] M Alenza DP Tabanera E Peña L Inflammatory mammary carcinoma in dogs: 33 cases (1995–1999). J Am Vet Med Assoc. (2001) 219:1110–14. 10.2460/javma.2001.219.1110 11700710

[90] Peña L Perez-Alenza MD Rodriguez-Bertos A Nieto A. Canine inflammatory mammary carcinoma: histopathology, immunohistochemistry and clinical implications of 21 cases. Breast Cancer Res Treat. (2003) 78:141–8. 10.1023/A:1022991802116 12725414

[91] Kim T Lau J Erban J. Lack of uniform diagnostic criteria for inflammatory breast cancer limits interpretation of treatment outcomes: a systematic review. Clin Breast Cancer. (2006) 7:386–95. 10.3816/CBC.2006.n.055 17239263

[92] Giordano SH Hortobagyi GN. Inflammatory breast cancer: clinical progress and the main problems that must be addressed. Breast Cancer Res. (2003) 5:284–88. 10.1186/bcr608 14580242 PMC314400

[93] Clemente M De Andres P Peña L Perez-Alenza M. Survival time of dogs with inflammatory mammary cancer treated with palliative therapy alone or palliative therapy plus chemotherapy. Vet Record. (2009) 165:78–81. 10.1136/vetrec.165.3.78 19617612

[94] Gilbertson S Kurzman I Zachrau R Hurvitz A Black M. Canine mammary epithelial neoplasms: biologic implications of morphologic characteristics assessed in 232 dogs. Vet Pathol. (1983) 20:127–42. 10.1177/030098588302000201 6836870

[95] Rosol TJ Tannehill-Gregg SH LeRoy BE Mandl S Contag CH. Animal models of bone metastasis. Cancer. (2003) 97:748–57. 10.1002/cncr.11150 12548572

[96] O'Shaughnessy J. Extending survival with chemotherapy in metastatic breast cancer. Oncologist. (2005) 10:20–9. 10.1634/theoncologist.10-90003-20 16368868

[97] Sleeckx N de Rooster H Veldhuis Kroeze E van Ginneken C van Brantegem L. Canine mammary tumours, an overview. Reprod Domest Anim. (2011) 46:1112–31. 10.1111/j.1439-0531.2011.01816.x 21645126

[98] Hellmen E Bergstrom R Holmberg L Spangberg IB Hansson K Lindgren A. Prognostic factors in canine mammary tumors: a multivariate study of 202 consecutive cases. Vet Pathol. (1993) 30:20–7. 10.1177/030098589303000103 8442324

[99] Pérez Alenza MD Peña L Nieto AI Castaño M. Clinical and pathological prognostic factors in canine mammary tumors. Ann Ist Super Sanita. (1997) 33:581–5. 9616968

[100] Sorenmo KU Kristiansen VM Cofone MA Shofer FS Breen A-M Langeland M. Canine mammary gland tumours; a histological continuum from benign to malignant; clinical and histopathological evidence. Vet Comp Oncol. (2009) 7:162–72. 10.1111/j.1476-5829.2009.00184.x 19691645

[101] Alenza MP Pena L del Castillo N Nieto A Factors influencing the incidence and prognosis of canine mammary tumours. J Small Anim Pract. (2000) 41:287–91. 10.1111/j.1748-5827.2000.tb03203.x 10976622

[102] Ferreira E Bertagnolli A Cavalcanti M Schmitt F Cassali G. The relationship between tumour size and expression of prognostic markers in benign and malignant canine mammary tumours. Vet Comp Oncol. (2009) 7:230–5. 10.1111/j.1476-5829.2009.00193.x 19891693

[103] Chang S-C Chang C-C Chang T-J Wong M-L. Prognostic factors associated with survival two years after surgery in dogs with malignant mammary tumors: 79 cases (1998–2002). J Am Vet Med Assoc. (2005) 227:1625–9. 10.2460/javma.2005.227.1625 16313041

[104] Philibert JC Snyder PW Glickman N Glickman LT Knapp DW Waters DJ. Influence of host factors on survival in dogs with malignant mammary gland tumors. J Vet Int Med. (2003) 17:102–6. 10.1111/j.1939-1676.2003.tb01330.x 12564734

[105] Karayannopoulou M Kaldrymidou E Constantinidis T Dessiris A. Histological grading and prognosis in dogs with mammary carcinomas: application of a human grading method. J Comp Pathol. (2005) 133:246–52. 10.1016/j.jcpa.2005.05.003 16202421

[106] Nguyen F Peña L Ibisch C Loussouarn D Gama A Rieder N. Canine invasive mammary carcinomas as models of human breast cancer. part 1: natural history and prognostic factors. Breast Cancer ResTreat. (2018) 167:635–48. 10.1007/s10549-017-4548-2 29086231 PMC5807494

[107] Nieto A Peña L Pérez-Alenza MD Sánchez MA Flores JM Castaño M. Immunohistologic detection of estrogen receptor alpha in canine mammary tumors: clinical and pathologic associations and prognostic significance. Vet Pathol. (2000) 37:239–47. 10.1354/vp.37-3-239 10810988

[108] Queiroga F Alves A Pires I Lopes C. Expression of Cox-1 and Cox-2 in canine mammary tumours. J Comp Pathol. (2007) 136:177–85. 10.1016/j.jcpa.2007.01.010 17416236

[109] Lavalle G Bertagnolli A Tavares W Cassali G. Cox-2 expression in canine mammary carcinomas: correlation with angiogenesis and overall survival. Vet Pathol. (2009) 46:1275–80. 10.1354/vp.08-VP-0226-C-FL 19605908

[110] Pereira PD Lopes C Matos A Santos M Gärtner F Medeiros R COX-2 expression in canine normal and neoplastic mammary gland. J Comp Pathol. (2009) 140:247–53. 10.1016/j.jcpa.2008.12.005 19203768

[111] Queiroga FL Pires I Lobo L Lopes CS. The role of Cox-2 expression in the prognosis of dogs with malignant mammary tumours. Res Vet Sci. (2010) 88:441–5. 10.1016/j.rvsc.2009.10.009 19939424

[112] de las Mulas JM Millán Y Dios R. A prospective analysis of immunohistochemically determined estrogen receptor α and progesterone receptor expression and host and tumor factors as predictors of disease-free period in mammary tumors of the dog. Vet Pathol. (2005) 42:200–12. 10.1354/vp.42-2-200 15753474

[113] Pena LL Nieto AI Pérez-Alenza D Cuesta P Castano M. Immunohistochemical detection of Ki-67 and PCNA in canine mammary tumors: relationship to clinical and pathologic variables. J Vet Diagn Invest. (1998) 10:237–46. 10.1177/104063879801000303 9683072

[114] Sarli G Preziosi R Benazzi C Castellani G Marcato PS. Prognostic value of histologic stage and proliferative activity in canine malignant mammary tumors. J Vet Diagn Invest. (2002) 14:25–34. 10.1177/104063870201400106 12680640

[115] Klopfleisch R Lenze D Hummel M Gruber AD. Metastatic canine mammary carcinomas can be identified by a gene expression profile that partly overlaps with human breast cancer profiles. BMC Cancer. (2010) 10:618–29. 10.1186/1471-2407-10-618 21062462 PMC2994823

[116] Klopfleisch R Klose P Weise C Bondzio A Multhaup G Einspanier R. Proteome of metastatic canine mammary carcinomas: similarities to and differences from human breast cancer. J Proteome Res. (2010) 9:6380–91. 10.1021/pr100671c 20932060

[117] MacEwen E Patnaik A Harvey H Panko W. Estrogen receptors in canine mammary tumors. Cancer Res. (1982) 42:2255–9. 7074608

[118] Rutteman G Misdorp W Blankenstein M van den Brom. Oestrogen (ER) and progestin receptors (PR) in mammary tissue of the female dog: different receptor profile in non-malignant and malignant states. Br J Cancer. (1988) 58:594–9. 10.1038/bjc.1988.266 3219269 PMC2246835

[119] Pichon MF Broet P Magdelenat H Delarue JC Spyratos F Basuyau JP. Prognostic value of steroid receptors after long-term follow-up of 2257 operable breast cancers. Br J Cancer. (1996) 73:1545–51. 10.1038/bjc.1996.291 8664127 PMC2074541

[120] Lumachi F Brunello A Maruzzo M Basso U Basso SM. Treatment of estrogen receptor-positive breast cancer. Curr Med Chem. (2013) 20:596–604. 10.2174/092986713804999303 23278394

[121] Badowska-Kozakiewicz AM Patera J Sobol M Przybylski J. The role of oestrogen and progesterone receptors in breast cancer - immunohistochemical evaluation of oestrogen and progesterone receptor expression in invasive breast cancer in women. Contemp Oncol. (2015) 19:220–5. 10.5114/wo.2015.51826 26557763 PMC4631285

[122] Kim NH Lim HY Im KS Shin JI Kim HW Sur JH. Evaluation of clinicopathological characteristics and Oestrogen receptor gene expression in Oestrogen receptor-negative, progesterone receptor-positive canine mammary Carcinomas. J Comp Pathol. (2014) 151:42–50. 10.1016/j.jcpa.2014.04.001 24913515

[123] Dowsett M Houghton J Iden C Salter J Farndon J A'hern R. Benefit from adjuvant tamoxifen therapy in primary breast cancer patients according oestrogen receptor, progesterone receptor, EGF receptor and HER2 status. Annals of Oncol. (2006) 17:818–26. 10.1093/annonc/mdl016 16497822

[124] Osborne CK Schiff R Arpino G Lee AS Hilsenbeck V. Endocrine responsiveness: understanding how progesterone receptor can be used to select endocrine therapy. Breast. (2005) 14:458–65. 10.1016/j.breast.2005.08.024 16236516

[125] Yip CH Rhodes A. Estrogen and progesterone receptors in breast cancer. Future Oncol. (2014) 10:2293–301. 10.2217/fon.14.110 25471040

[126] Mainenti M Rasotto R Carnier P Zappulli V. Oestrogen and progesterone receptor expression in subtypes of canine mammary tumours in intact and ovariectomised dogs. Vet J. (2014) 202:62–8. 10.1016/j.tvjl.2014.06.003 24980810

[127] Millanta F Calandrella M Bari G Niccolini M Vannozzi I Poli A. Comparison of steroid receptor expression in normal, dysplastic, and neoplastic canine and feline mammary tissues. Res Vet Sci. (2005) 79:225–32. 10.1016/j.rvsc.2005.02.002 16054892

[128] Ross JS Fletcher JA Linette GP Stec J Clark E Ayers M. The Her-2/neu gene and protein in breast cancer 2003: biomarker and target of therapy. Oncologist. (2003) 8:307–25. 10.1634/theoncologist.8-4-307 12897328

[129] Zhao J Wu R Au A Marquez A Yu Y Shi Z. Determination of HER2 gene amplification by chromogenic *in situ* hybridization (CISH) in archival breast carcinoma. Mod Pathol. (2002) 15:657–65. 10.1038/modpathol.3880582 12065780

[130] Pauletti G Dandekar S Rong H Ramos L Peng H Seshadri R. Assessment of methods for tissue-based detection of the HER-2/neu alteration in human breast cancer: a direct comparison of fluorescence *in situ* hybridization and immunohistochemistry. J Clin Oncol. (2000) 18:3651–64. 10.1200/JCO.2000.18.21.3651 11054438

[131] Dutra A Granja N Schmitt F Cassali G. c-erbB-2 expression and nuclear pleomorphism in canine mammary tumors. Braz J Med Biol Res. (2004) 37:1673–81. 10.1590/S0100-879X2004001100013 15517084

[132] Hsu WL Huang HM Liao JW Wong ML Chang SC. Increased survival in dogs with malignant mammary tumours overexpressing HER-2 protein and detection of a silent single nucleotide polymorphism in the canine HER-2 gene. Vet J. (2009) 180:116–23. 10.1016/j.tvjl.2007.10.013 18061495

[133] Muhammadnejad A Keyhani E Mortazavi P Behjati F Haghdoost IS. Overexpression of her-2/neu in malignant mammary tumors; translation of clinicopathological features from dog to human. Asian Pac J Cancer Prev. (2012) 13:6415–21. 10.7314/APJCP.2012.13.12.6415 23464468

[134] Ahern T Bird R Bird A Wolfe L. Expression of the oncogene c-erbB-2 in canine mammary cancers and tumor-derived cell lines. Am J Vet Res. (1996) 57:693–6. 8723884

[135] Martín J de las Mulas Ordás J Millán Y Fernández-Soria V Ramón y Cajal S Oncogene HER-2 in canine mammary gland Carcinomas. Breast Cancer Research and Treat. (2003) 80:363–7. 10.1023/A:1024929730165 14503809

[136] Silva I Dias A Bertagnolli A Cassali G Ferreira E Analysis of EGFR and HER-2 expressions in ductal carcinomas *in situ* in canine mammary glands. Arquivo Brasileiro de Medicina Veterinária e Zootecnia. (2014) 66:763–8. 10.1590/1678-41626128

[137] Kaszak I Ruszczak A Kanafa S Kacprzak K Król M Jurka P. Current biomarkers of canine mammary tumors. Acta Veterinaria Scandinavica. (2018) 60:66–72. 10.1186/s13028-018-0417-1 30373614 PMC6206704

[138] Campos LC Silva JO Santos FS Araujo MR Lavalle GE Ferreira E. Prognostic significance of tissue and serum HER2 and MUC1 in canine mammary cancer. J Vet Diagn Invest. (2015) 27:531–5. 10.1177/1040638715592445 26179096

[139] Ressel L Puleio R Loria GR Vannozzi I Millanta F Caracappa S. HER-2 expression in canine morphologically normal, hyperplastic and neoplastic mammary tissues and its correlation with the clinical outcome. Res Vet Sci. (2013) 94:299–305. 10.1016/j.rvsc.2012.09.016 23141215

[140] Singer J Weichselbaumer M Stockner T Mechtcheriakova D Sobanov Y Bajna E. Comparative oncology: ErbB-1 and ErbB-2 homologues in canine cancer are susceptible to cetuximab and trastuzumab targeting. Mol Immunol. (2012) 50:200–9. 10.1016/j.molimm.2012.01.002 22424313 PMC3318186

[141] Masuda H Zhang D Bartholomeusz C Doihara H Hortobagyi GN Ueno NT. Role of epidermal growth factor receptor in breast cancer. Breast Cancer Res Treat. (2012) 136:331–45. 10.1007/s10549-012-2289-9 23073759 PMC3832208

[142] Carvalho MI Guimaraes MJ Pires I Prada J Silva-Carvalho R Lopes C. EGFR and microvessel density in canine malignant mammary tumours. Res Vet Sci. (2013) 95:1094–9. 10.1016/j.rvsc.2013.09.003 24091029

[143] Gama A Gärtner F Alves A Schmitt F. Immunohistochemical expression of Epidermal Growth Factor Receptor (EGFR) in canine mammary tissues. Res Vet Sci. (2009) 87:432–37. 10.1016/j.rvsc.2009.04.016 19464036

[144] Guimaraes M Carvalho M Pires I Prada J Gil AG Lopes C. Concurrent expression of cyclo-oxygenase-2 and epidermal growth factor receptor in canine malignant mammary tumours. J Comp Pathol. (2014) 150:27–34. 10.1016/j.jcpa.2013.07.005 24060154

[145] Uva P Aurisicchio L Watters J Loboda A Kulkarni A Castle J. Comparative expression pathway analysis of human and canine mammary tumors. BMC Genomics. (2009) 10:135–55. 10.1186/1471-2164-10-135 19327144 PMC2670324

[146] Depowski PL Rosenthal SI Ross JS. Loss of expression of the PTEN gene protein product is associated with poor outcome in breast cancer. Mod Pathol. (2001) 14:672–6. 10.1038/modpathol.3880371 11454999

[147] Saal LH Johansson P Holm K Gruvberger-Saal SK She Q-B Maurer M. Poor prognosis in carcinoma is associated with a gene expression signature of aberrant PTEN tumor suppressor pathway activity. Proc Natl Acad Sci USA. (2007) 104:7564–9. 10.1073/pnas.0702507104 17452630 PMC1855070

[148] Galanis A Pappa A Giannakakis A Lanitis E Dangaj D Sandaltzopoulos R. Reactive oxygen species and HIF-1 signalling in cancer. Cancer Lett. (2008) 266:12–20. 10.1016/j.canlet.2008.02.028 18378391

[149] Castaneda CA Cortes-Funes H Gomez HL Ciruelos EM. The phosphatidyl inositol 3-kinase/AKT signaling pathway in breast cancer. Cancer Metastasis Rev. (2010) 29:751–9. 10.1007/s10555-010-9261-0 20922461

[150] Dillon R White D Muller W. The phosphatidyl inositol 3-kinase signaling network: implications for human breast cancer. Oncogene. (2007) 26:1338–45. 10.1038/sj.onc.1210202 17322919

[151] Pinho SS Carvalho S Cabral J Reis CA Gärtner F. Canine tumors: a spontaneous animal model of human carcinogenesis. Transl Res. (2012) 159:165–72. 10.1016/j.trsl.2011.11.005 22340765

[152] Ressel L Millanta F Caleri E Innocenti V Poli A. Reduced PTEN protein expression and its prognostic implications in canine and feline mammary tumors. Vet Pathol. (2009) 46:860–8. 10.1354/vp.08-VP-0273-P-FL 19429983

[153] Qiu C Lin D Wang J Li C Deng G. Expression and significance of PTEN and VEGF in canine mammary gland tumours. Vet Res Commun. (2008) 32:463–72. 10.1007/s11259-008-9049-7 18461467

[154] Gama A Paredes J Gartner F Alves A Schmitt F. Expression of E-cadherin, P-cadherin and beta-catenin in canine malignant mammary tumours in relation to clinicopathological parameters, proliferation and survival. Vet J. (2008) 177:45–53. 10.1016/j.tvjl.2007.05.024 17631398

[155] Nowak M Madej JA Dziegiel P. Expression of E-cadherin, beta-catenin and Ki-67 antigen and their reciprocal relationships in mammary adenocarcinomas in bitches. Folia Histochem Cytobiol. (2007) 45:233–8. 17951173

[156] Matos AJ Lopes C Carvalheira J Santos M Rutteman GR Gartner F. E-cadherin expression in canine malignant mammary tumours: relationship to other clinico-pathological variables. J Comp Pathol. (2006) 134:182–9. 10.1016/j.jcpa.2005.10.004 16545841

[157] Li Z Yin S Zhang L Liu W Chen B. Prognostic value of reduced E-cadherin expression in breast cancer: a meta-analysis. Oncotarget. (2017) 8:16445. 10.18632/oncotarget.14860 28147315 PMC5369975

[158] Paredes J Correia AL Ribeiro AS Albergaria A Milanezi F Schmitt FC. P-cadherin expression in breast cancer: a review. Breast Cancer Res. (2007) 9:214–26. 10.1186/bcr1774 18001487 PMC2242663

[159] Velculescu VE El-Deiry WS. Biological and clinical importance of the p53 tumor suppressor gene. Clin Chem. (1996) 42:858–68. 10.1093/clinchem/42.6.858 8665676

[160] Pan Y Yuan Y Liu G Wei Y. P53 and Ki-67 as prognostic markers in triple-negative breast cancer patients. PLoS ONE. (2017) 12:e0172324. 10.1371/journal.pone.0172324 28235003 PMC5325264

[161] Chu LL Rutteman GR Kong JM Ghahremani M Schmeing M Misdorp W. Genomic organization of the canine p53 gene and its mutational status in canine mammary neoplasia. Breast Cancer Res Treat. (1998) 50:11–25. 10.1023/A:1006010526813 9802616

[162] Kim K Chie EK Han W Noh D-Y Park I Oh D-Y. Prognostic value of p53 and bcl-2 expression in patients treated with breast conservative therapy. J Korean Med Sci. (2010) 25:235–9. 10.3346/jkms.2010.25.2.235 20119576 PMC2811290

[163] Lee C-H Kim W-H Lim J-H Kang M-S Kim D-Y Kweon O-K. Mutation and overexpression of p53 as a prognostic factor in canine mammary tumors. J Vet Sci. (2004) 5:63–70. 10.4142/jvs.2004.5.1.63 15028887

[164] Beenken SW Grizzle WE Crowe DR Conner MG Weiss HL Sellers MT. Molecular biomarkers for breast cancer prognosis: coexpression of c-erbB-2 and p53. Ann Surg. (2001) 233:630–8. 10.1097/00000658-200105000-00006 11323501 PMC1421302

[165] Lee CH Kweon OK. Mutations of p53 tumor suppressor gene in spontaneous canine mammary tumors. J Vet Sci. (2002) 3:321–6. 10.4142/jvs.2002.3.4.321 12819382

[166] Wakui S Muto T Yokoo K Yokoo R Takahashi H Masaoka T. Prognostic status of p53 gene mutation in canine mammary carcinoma. Anticancer Res. (2001) 21:611–6. 11299814

[167] Queiroga FL Raposo T Carvalho MI Prada J Pires I. Canine mammary tumours as a model to study human breast cancer: most recent findings. In Vivo. (2011) 25:455–65. 21576423

[168] Abadie J Nguyen F Loussouarn D Peña L Gama A Rieder N. Canine invasive mammary carcinomas as models of human breast cancer. Part 2: immunophenotypes and prognostic significance. Breast Cancer Res Treat. (2018) 167:459–68. 10.1007/s10549-017-4542-8 29063312 PMC5790838

[169] Gama A Alves A Schmitt F. Identification of molecular phenotypes in canine mammary carcinomas with clinical implications: application of the human classification. Virchows Arch. (2008) 453:123–32. 10.1007/s00428-008-0644-3 18677512

[170] Liu D Xiong H Ellis AE Northrup NC Rodriguez CO O'Regan RM. Molecular homology and difference between spontaneous Canine mammary cancer and human breast cancer. Cancer Res. (2014) 74:5045–56. 10.1158/0008-5472.CAN-14-0392 25082814 PMC4167563

[171] Easton DF Pooley KA Dunning AM Pharoah PD Thompson D Ballinger DG. Genome-wide association study identifies novel breast cancer susceptibility loci. Nature. (2007) 447:1087. 10.1038/nature05887 17529967 PMC2714974

[172] Rousseau J Têtu B Caron D Malenfant P Cattaruzzi P Audette M. RCAS1 is associated with ductal breast cancer progression. Biochem Biophys Res Commun. (2002) 293:1544–9. 10.1016/S0006-291X(02)00401-1 12054692

[173] Meijers-Heijboer H Wasielewski M Wagner A Hollestelle A Elstrodt F van den Bos R. The CHEK2 1100delC mutation identifies families with a hereditary breast and colorectal cancer phenotype. Am J Hum Genet. (2003) 72:1308–14. 10.1086/375121 12690581 PMC1180284

[174] Consortium C-BC Low-penetrance susceptibility to breast cancer due to CHEK2^*^ 1100delC in noncarriers of BRCA1 or BRCA2 mutations. Nat Genetics. (2002) 31:55 10.1038/ng879 11967536

[175] Walsh T King M-C. Ten genes for inherited breast cancer. Cancer Cell. (2007) 11:103–5. 10.1016/j.ccr.2007.01.010 17292821

[176] Pharoah PD Dunning AM Ponder BA Easton DF. Association studies for finding cancer-susceptibility genetic variants. Nat Rev Cancer. (2004) 4:850. 10.1038/nrc1476 15516958

[177] Venkitaraman AR. Cancer susceptibility and the functions of BRCA1 and BRCA2. Cell. (2002) 108:171–82. 10.1016/S0092-8674(02)00615-3 11832208

[178] Yoshikawa Y Morimatsu M Ochiai K Ishiguro-Oonuma T Wada S Orino K. Reduced canine BRCA2 expression levels in mammary gland tumors. BMC Vet Res. (2015) 11:159–64. 10.1186/s12917-015-0483-9 26202431 PMC4512014

[179] Söderlund K Skoog L Fornander T Askmalm MS. The BRCA1/BRCA2/Rad51 complex is a prognostic and predictive factor in early breast cancer. Radiother Oncol.(2007) 84:242–51. 10.1016/j.radonc.2007.06.012 17707537

[180] Ochiai K Morimatsu M Tomizawa N Syuto B. Cloning and sequencing full length of canine Brca2 and Rad51 cDNA. J Vet Med Sci. (2001) 63:1103–8. 10.1292/jvms.63.1103 11714026

[181] Martin A Greshock J Rebbeck T Weber B Allele frequencies of cytokine gene polymorphisms in Caucasians and African Americans. Am J Hum Genet. (2000) 67:318–9.

[182] Ford D Easton D Stratton M Narod S Goldgar D Devilee P Chang-Claude J, Genetic heterogeneity and penetrance analysis of the BRCA1 and BRCA2 genes in breast cancer families. Am J Hum Genet. (1998) 62:676–89. 10.1086/301749 9497246 PMC1376944

[183] M.-King C Marks JH Mandell JB Breast and ovarian cancer risks due to inherited mutations in BRCA1 and BRCA2. Science. (2003) 302:643–6. 10.1126/science.1088759 14576434

[184] Struewing JP Hartge P Wacholder S Baker SM Berlin M McAdams M. The risk of cancer associated with specific mutations of BRCA1 and BRCA2 among Ashkenazi Jews. N Engl J Med. (1997) 336:1401–8. 10.1056/NEJM199705153362001 9145676

[185] Couch FJ Weber BL. Mutations and polymorphisms in the familial early-onset breast cancer (BRCA1) gene. Hum Mutat. (1996) 8:8–18. 10.1002/humu.1380080102 8807330

[186] Walsh T Casadei S Coats KH Swisher E Stray SM Higgins J. Spectrum of mutations in BRCA1, BRCA2, CHEK2, and TP53 in families at high risk of breast cancer. JAMA. (2006) 295:1379–88. 10.1001/jama.295.12.1379 16551709

[187] Chang J Elledge RM. Clinical management of women with genomic BRCA1 and BRCA2 mutations. Breast Cancer Res Treat. (2001) 69:101–13. 10.1023/A:1012203917104 11759816

[188] Bieche I Nogues C Lidereau R. Overexpression of BRCA2 gene in sporadic breast tumours. Oncogene. (1999) 18:5232. 10.1038/sj.onc.1202903 10498873

[189] Thompson ME Jensen RA Obermiller PS Page DL Holt JT. Decreased expression of BRCA1 accelerates growth and is often present during sporadic breast cancer progression. Nat Genetics. (1995) 9:444. 10.1038/ng0495-444 7795653

[190] Majdak-Paredes E Fatah F. Hereditary breast cancer syndromes and clinical implications. J Plast Reconstr Aesthet Surg. (2009) 62:181–9. 10.1016/j.bjps.2008.07.012 19027378

[191] Rivera P Melin M Biagi T Fall T Häggström J Lindblad-Toh K. Mammary tumor development in dogs is associated with BRCA1 and BRCA2. Cancer Res. (2009) 69:8770–4. 10.1158/0008-5472.CAN-09-1725 19887619

[192] Klopfleisch R Gruber AD. Increased expression of BRCA2 and RAD51 in lymph node metastases of canine mammary adenocarcinomas. Vet Pathol. (2009) 46:416–22. 10.1354/vp.08-VP-0212-K-FL 19176491

[193] Nieto A Pérez-Alenza MD Del Castillo N Tabanera E Castaño M Peña L. BRCA1 expression in Canine mammary Dysplasias and tumours: relationship with prognostic variables. J Comp Pathol. (2003) 128:260–8. 10.1053/jcpa.2002.0631 12834609

[194] Elmore JG Armstrong K Lehman CD Fletcher SW. Screening for breast cancer. JAMA. (2005) 293:1245–56. 10.1001/jama.293.10.1245 15755947 PMC3149836

[195] Morgan MP Cooke MM McCarthy GM. Microcalcifications associated with breast cancer: an epiphenomenon or biologically significant feature of selected tumors? J Mammary Gland Biol Neoplasia. (2005) 10:181–7. 10.1007/s10911-005-5400-6 16025224

[196] Mohammed SI Meloni GB Parpaglia MP Marras V Burrai GP Meloni F. Mammography and ultrasound imaging of preinvasive and invasive canine spontaneous mammary cancer and their similarities to human breast cancer. Cancer Prev Res. (2011) 4:1790–8. 10.1158/1940-6207.CAPR-11-0084 21803985

[197] Andrew Novosad C. Principles of treatment for mammary gland tumors. Clin Techniq Small Anim Practice. (2003) 18:107–9. 10.1053/svms.2003.36625 12831071

[198] Stratmann N Failing K Richter A Wehrend A. Mammary tumor recurrence in bitches after regional mastectomy. Vet Surg. (2008) 37:82–6. 10.1111/j.1532-950X.2007.00351.x 18199060

[199] Kristiansen VM Peña L Díez Córdova L Illera JC Skjerve E Breen AM. Effect of Ovariohysterectomy at the time of tumor removal in dogs with mammary carcinomas: a randomized controlled trial. J Vet Int Med. (2016) 30:230–41. 10.1111/jvim.13812 26687731 PMC4913665

[200] Karayannopoulou M Kaldrymidou E Constantinidis T Dessiris A. Adjuvant post-operative chemotherapy in bitches with mammary cancer. J Vet Med Series A. (2001) 48:85–96. 10.1046/j.1439-0442.2001.00336.x 11315572

[201] Kitchell B Fidel J Tamoxifen as a potential therapy for canine mammary carcinoma, Proceedings. Vet Can Soc. (1992) 91:91–4.

[202] Morris JS Dobson JM Bostock DE. Use of tamoxifen in the control of canine mammary neoplasia. Vet Rec. (1993) 133:539–42. 10.1136/vr.133.22.539 8116156

[203] Tavares WLF Lavalle GE Figueiredo MS Souza AG Bertagnolli AC Viana FAB. Evaluation of adverse effects in tamoxifen exposed healthy female dogs. Acta Vet Scand. (2010) 52:67–74. 10.1186/1751-0147-52-67 21176231 PMC3022551

[204] Day C-P Merlino G van Dyke T. Preclinical mouse cancer models: a maze of opportunities and challenges. Cell. (2015) 163:39–53. 10.1016/j.cell.2015.08.068 26406370 PMC4583714

[205] London CA Hannah AL Zadovoskaya R Chien MB Kollias-Baker C Rosenberg M. Phase I dose-escalating study of SU11654, a small molecule receptor tyrosine kinase inhibitor, in dogs with spontaneous malignancies. Clin Cancer Res. (2003) 9:2755–68. 12855656

[206] Mendel DB Laird AD Xin X Louie SG Christensen JG Li G. *In vivo* antitumor activity of SU11248, a novel tyrosine kinase inhibitor targeting vascular endothelial growth factor and platelet-derived growth factor receptors: determination of a pharmacokinetic/pharmacodynamic relationship. Clin Cancer Res. (2003) 9:327–37. 12538485

[207] Palmu S Söderström K Quazi K Isola J Salminen E. Expression of C-KIT and HER-2 tyrosine kinase receptors in poor-prognosis breast cancer. Anticancer Res. (2002) 22:411–4. 12017324

[208] de Jong JS van Diest PJ van Der Valk P Baak JP. Expression of growth factors, growth inhibiting factors, and their receptors in invasive breast cancer. I: an inventory in search of autocrine and paracrine loops. J Pathol. (1998) 184:44–52. 9582526 10.1002/(SICI)1096-9896(199801)184:1<44::AID-PATH984>3.0.CO;2-H

[209] de Jong JS van Diest PJ van Der Valk P Baak JP. Expression of growth factors, growth-inhibiting factors, and their receptors in invasive breast cancer. II: Correlations with proliferation and angiogenesis. J Pathol. (1998) 184:53–7. 9582527 10.1002/(SICI)1096-9896(199801)184:1<53::AID-PATH6>3.0.CO;2-7

[210] Kubo K Matsuyama S Katayama K Tsutsumi C Yonezawa K Shimada T. Frequent expression of the c-kit proto-oncogene in canine malignant mammary tumor. J Vet Med Sci. (1998) 60:1335–40. 10.1292/jvms.60.1335 9879535

[211] London CA Malpas PB Wood-Follis SL Boucher JF Rusk AW Rosenberg MP. Multi-center, placebo-controlled, double-blind, randomized study of oral Toceranib phosphate (SU11654), a receptor Tyrosine kinase inhibitor, for the treatment of dogs with recurrent (either local or distant) mast cell tumor following surgical excision. Clin Cancer Res. (2009) 15:3856. 10.1158/1078-0432.CCR-08-1860 19470739

[212] Elgebaly A Menshawy A El Ashal G Osama O Ghanem E Omar A. Sunitinib alone or in combination with chemotherapy for the treatment of advanced breast cancer: a systematic review and meta-analysis. Breast Dis. (2016) 36:91–101. 10.3233/BD-160218 27612040

